# Mid- and long-term responses of land snail communities to the intensification of mountain hay meadows management

**DOI:** 10.1186/s12862-022-01972-4

**Published:** 2022-02-15

**Authors:** Gerard Martínez-De León, Lauriane Dani, Aline Hayoz-Andrey, Ségolène Humann-Guilleminot, Raphaël Arlettaz, Jean-Yves Humbert

**Affiliations:** Division of Conservation Biology, Institute of Ecology and Evolution, University of Bern, Baltzerstrasse 6, 3012 Bern, Switzerland

**Keywords:** Alps, Conservation, Fertilisation, Gastropod, Grassland

## Abstract

**Background:**

Species-rich semi-natural grasslands are impacted by the severe land-use changes that are affecting mountain regions, compromising their high biodiversity value. In particular, sprinkler irrigation and increased fertilisation stimulate vegetation growth, modifying and homogenising habitat conditions for ground-dwelling invertebrates. Among them, land snails have been largely understudied despite their commonness and vulnerability to small-scale habitat alteration. This study investigated the mid- and long-term responses of land snail communities to management intensification of montane and subalpine hay meadows. Mid-term effects were studied using a randomised block design experiment, mimicking an intensification gradient with different levels of irrigation and fertilisation applied during 5 years. Long-term effects were examined relying on an observational approach that consisted in comparing snail communities in meadows managed intensively for > 20 years with those from the 5-year experimental module.

**Results:**

We show that management intensification initially boosts snail densities, but erodes species richness by − 35% in intensively-managed meadows in the long term. Contrary to our expectations, drought-tolerant (xerophilous) snails benefitted from grassland intensification, whereas mesophilous species accounted for most species losses due to intensification in the long run, indicating that the latter may be especially sensitive to the hostile microclimate conditions abruptly prevailing in a meadow after mowing. Soil pH was also a principal determinant of land snail occurrence, with almost no specimen recorded in acidic meadows (pH < 5.5), while plant diversity favoured overall snail abundance.

**Conclusions:**

Despite the fact that xerophilous snails appear tolerant to management intensification, we found that several drought-sensitive species are lost in the long term. We conclude that the preservation of species-rich land snail communities in mountain hay meadows requires the conservation and restoration of low-input grasslands on basic soils for preventing further species losses of gastropod fauna.

**Supplementary Information:**

The online version contains supplementary material available at 10.1186/s12862-022-01972-4.

## Background

Semi-natural grasslands are among the most specious habitats of temperate biomes, harbouring at site scale many more plant and arthropod taxa than the native habitat that would naturally develop at their place in the absence of human management [1–4]. Nevertheless, rising socio-economic pressures to increase yield (forage production) and to optimize agricultural labour are triggering widespread land-use changes that cause a collapse of traditionally-managed biodiversity-rich grasslands [5, 6]. Farmland intensification aims to obtain higher yields mainly through the addition of fertilisers [7], favouring fast-growing plants and generating a more homogeneous and shaded understory [8]. Intensification in mountain regions is mostly restricted to sites where access to agricultural machinery is possible, while land abandonment follows in principle the cessation of farming in difficult terrain and naturally less productive areas [9]. Because of the constraints imposed by their complex topography, mountain meadows are generally smaller, and less frequently fertilised and mown than lowland grasslands [2, 3, 10], meaning that the impact of intensification in these areas is potentially milder than at low elevation.

Despite being key components of grassland ecosystem functioning (e.g. [11]), invertebrates, contrary to plants, have been little investigated for assessing the impact of mountain grassland intensification [12]. Invertebrate taxa show different responses to management intensification, mostly according to their life-history traits: ectothermic heat-demanding taxa are ecologically especially vulnerable, i.e. sensitive to intensification (e.g. orthopterans; [13]), whereas taxa relying on an abundant phytomass can thrive under a more intensive management regime (e.g. carabids, leafhoppers and spiders; [14–16]). Yet, responses remain hard to predict for some taxa whose various life-history traits tend to react in opposite directions under the action of a given driver. Land snails are a good example. They are both abundant and functionally important grass-dwelling invertebrates, this owing to their role as detritivores [17] and prey for the upper trophic levels of the food chain [18, 19]. Their general extremely low active mobility and the high specialization of many species [20–22] might render them particularly sensitive to grassland intensification, although their small body size and activity taking place mainly at ground level [23] could confer them an enhanced tolerance to vegetation disturbances (e.g. mowing) compared to other invertebrates [24, 25]. Understanding the response of land snails to farming intensification would represent an asset to better inform conservation strategies aiming at conciliating the preservation of open-land biodiversity and nature-friendly agricultural production.

The study was carried out in Valais (SW Swiss Alps), a region characterized by warm and dry summers where the management of montane and subalpine hay meadows was traditionally achieved via irrigation by open water channels (gravitation) and fertilisation with solid organic manure, while modern farming mostly involves irrigation with sprinklers and slurry inputs. Since snail activity is highly dependent on moisture conditions [26–28], we hypothesised that farming intensification would promote higher snail densities due to greater water and fertiliser inputs that entail a denser sward and induce cooler and wetter conditions at ground level [13, 29]. We also predicted that open-land, drought-tolerant (i.e. xerophilous) snail species would be well represented in low-input grasslands, but would gradually become less frequent with increasing management intensity and a long exposure to new management modes [30]. In effect, it has been suggested that sensitive species may show a delayed response to newly generated unsuitable environmental conditions, becoming less abundant over time and eventually disappearing locally [31].

In order to unravel the impact of grassland management intensification on land snail communities over time, we relied on two approaches. The mid-term effects of intensification (after 5 years) were investigated in a first module, with a randomised block design experiment, by applying different levels of irrigation and fertilisation that mimic an increasing gradient of farming intensity. To our knowledge, this is the first genuine experiment (random allocation of treatments to plots) that has tested the effects of grassland management intensification on land snails. In a second module, the long-term effects of intensification were investigated within an observational framework, by comparing snail communities collected from intensively-managed meadows (> 20 years of intensive management) with those stemming from the above mid-term experimental module. The combination of both modules offers a comprehensive analysis of the effects of grassland intensification on land snails, thus providing solid support for the conservation and restoration of hay meadow biodiversity. Finally, several environmental variables were recorded in the intensively-managed meadows of the observational module so as to (1) identify the key environmental factors driving the variation among meadow snail assemblages; and (2) disentangle the contribution of natural and anthropogenic factors in shaping their composition.

## Methods

### Study area and experimental design

Study sites were located in the canton of Valais, in the inner Swiss Alps, between 880 and 1768 m a.s.l. (Additional file 1: Appendix S1). Land snails from the experimental module were sampled in 2015 at eleven meadows scattered across the study area. These had to be managed extensively for at least 10 years before the onset of the experiment in 2010, with no or very low levels of fertilisation and irrigation and only a single cut per year. Within each meadow, three management treatments and a control were randomly allocated to 20 m diameter plots, with at least 5 m buffer between adjacent plots. The control plot received no input while the other three plots received low, medium or high inputs of fertiliser and water, with respectively 1/3, 2/3 or 3/3 of a quantity that had been estimated necessary to achieve maximum hay yield at a given locality (for further details on the experimental design see Appendix A in [14]). Snail abundance and species richness did not differ among plots before the different management treatments were applied [32]. All plots were mown twice a year, except control plots that were mown only once to mimic local standards for extensively-managed meadows. Weekly irrigation amounted to 10, 20 and 30 mm in, respectively, low, medium and high input plots. These plots were irrigated from mid-May until early September, except under heavy rainfall (> 20 mm water during the previous week). Fertiliser consisted of dried organic manure NPK pellets (MEOC SA, 1906 Charrat, Switzerland) and mineral potassium sulphate (K_2_SO_4_) dissolved into water so as to reach the nutrient content and viscosity of standard farm slurry.

Snails from the observational module were collected in the year 2019 from 39 meadows at thirteen different sites. These meadows had a minimum area of 0.2 ha and had to be managed intensively (i.e. frequently fertilised with solid or liquid manure, mown at least twice a year, and often used as pasture in autumn) for at least 20 years. Different types of fertiliser (manure or slurry) were usually alternated haphazardly between years depending on local farming mode and constrains so that it was not possible to incorporate this factor in the analyses. Other management practices (e.g. autumn grazing, number of grass cuts, technique of mowing, historical management, etc.) were similar between study sites and were thus not accounted for in our models.

### Data collection

Snails present in the soil and the litter layer were collected from soil cores. Following the Swiss Biodiversity Monitoring (BDM) protocol for terrestrial molluscs [33], eight soil samples of 125 cm^2^ area and 5 cm depth each were extracted after the first hay cut and pooled afterwards into a 5 dm^3^ sample. These samples were then processed to separate the shells from the soil fraction, using a set of sieves (mesh sizes of 10, 2 and 0.7 mm) and then examined visually. Fresh shells were identified under the binocular microscope, according to Boschi [34] and Hausser [35]. The same sampling method was adopted in both modules, allowing for quantitative comparison of community composition.

The moisture preferences of different species of snails were extracted from an extensive trait database [23]. Species were categorised into xerophilous, mesophilous or hygrophilous depending on whether they showed highest affinity for dry, moist or wet soils, respectively. Besides, a community weighted mean (CWM) of moisture was calculated by weighing the moisture value of each species by its relative abundance in a given meadow, and then summing these weighed values. These species-specific moisture values were calculated following the methodology of Astor et al. [36]. Information on the regional Red-List status for every species was extracted from Rüetschi et al. [37].

In both modules, eight soil subsamples of 10 cm depth were obtained from each plot after the first cut and pooled into a 1 kg sample. Soil samples were then dried at 50 °C and sieved with a 2 mm mesh size. Soil pH was measured with a pH meter, after diluting 20 g per sample into 50 mL H_2_O.

In the observational module, vegetation-related variables were extracted from surveys conducted in two randomly allocated subplots of 2 × 4 m distant by 8 m, as in van Klink et al. [38]. In addition, several variables related to topography, soil, local landscape and agricultural management were collected. All measured variables and their description are presented in Additional file 2: Appendix S2.

### Statistical analyses

Land snail communities from the experimental and observational modules were compared to investigate the long-term effects of grassland management practices, using agricultural management intensity of each meadow (i.e. extensive, mid-term intensified, long-term intensified) as a fixed effect in the analyses. The extensive and mid-term intensified treatments were taken from the control and high management intensity plots of the experimental module, respectively, while the long-term intensified treatment included the meadows of the observational module. Generalised linear mixed models (GLMM) with Poisson error distribution were used with snail density and species richness (total, and for each moisture preference group), as well as the number of red-listed species as response variables. Linear mixed models (LMM) were adopted to analyse the response of the CWM of soil moisture optima and Pielou’s evenness index (J = H/ln(S), where H = the Shannon–Wiener index and S = number of species in the sample). In the models fitting a Gaussian error distribution, p-values were obtained using the *lmerTest* package [39]. Study site was set as a random factor in all models. An observation-level random effect was added in case overdispersion had to be handled [40]. Model-based community analyses were carried out with the function *manyglm* in the package *mvabund* (v. 4.1.3; [41]). This function fits a generalised linear model to a matrix of species abundances by performing univariate models to each taxon and then summing the test statistics [42]. Given the high amount of species occurring at few sites, we conducted the analyses only with those species present in at least nine samples from both the experimental and observational modules. Statistical significance was assessed with likelihood-ratio test statistics resampled 999 times with the PIT-trap method (function *anova.manyglm*). All the aforementioned analyses were done on a subset of samples delimited by a cut-off value of soil pH > 6, as this factor was strongly limiting snail communities in the study area (see ‘Results’). After accounting for this limitation of soil pH, 23 replicates from long-term intensified meadows, 10 replicates from mid-term intensified plots and 9 replicates from extensive plots were analysed.

In the experimental module, management treatments were converted into a continuous management intensity gradient variable (0, 1, 2, 3, respectively) ranging from no input to high-input, and then treated as a fixed effect. Besides snails-related response variables, the impact of management intensification on soil pH was also analysed with LMM. Community analyses for this module alone were done with the species present in at least nine samples.

In the observational module, a model selection approach was used to identify the most influential environmental variables for snail communities in the study system. Before model selection, correlations between covariates were assessed; in case two explanatory variables correlated (Spearman correlation coefficient > 0.7), the variable of more direct biological significance was kept [43, 44]. In a first step, a pre-selection of explanatory variables was done from the full initial set. For this purpose, univariate GLMMs were fitted for each standardised explanatory variable (mean = 0, standard deviation = 1), and only those statistically significant with *P* < 0.05 were retained. In a second step, all possible models (i.e. combinations of explanatory variables) were fitted and ranked using Akaike’s Information Criterion corrected for small sample sizes (AICc, Additional file 6: Appendix S6) with the function *dredge* in the package MuMIn (version 1.43.6; [45]). In case several models had similar support, a subset of top models within Δ AICc < 6 (following the recent suggestion by [43]) was selected for full model averaging with the function *model.avg* of the same package. The variables influencing the number of red-listed species were assessed with univariate GLMMs, as these species were too scarce at the study sites to be able to perform model selection. Models always satisfied the underlying assumptions of normal distribution of residuals and homoscedasticity. All the analyses were performed with the software R (v. 4.0.0; [46]).

## Results

Across all study sites, 8983 fresh shells were collected, belonging to 38 different species of land snails. 9 species were classified as xerophilous (23.7%), 22 as mesophilous (57.9%) and 6 as hygrophilous (15.8%; see Additional file 3: Appendix S3 for the complete species list).

### Long-term effects of grassland management intensification on land snail communities (both modules)

**Fig. 1:**
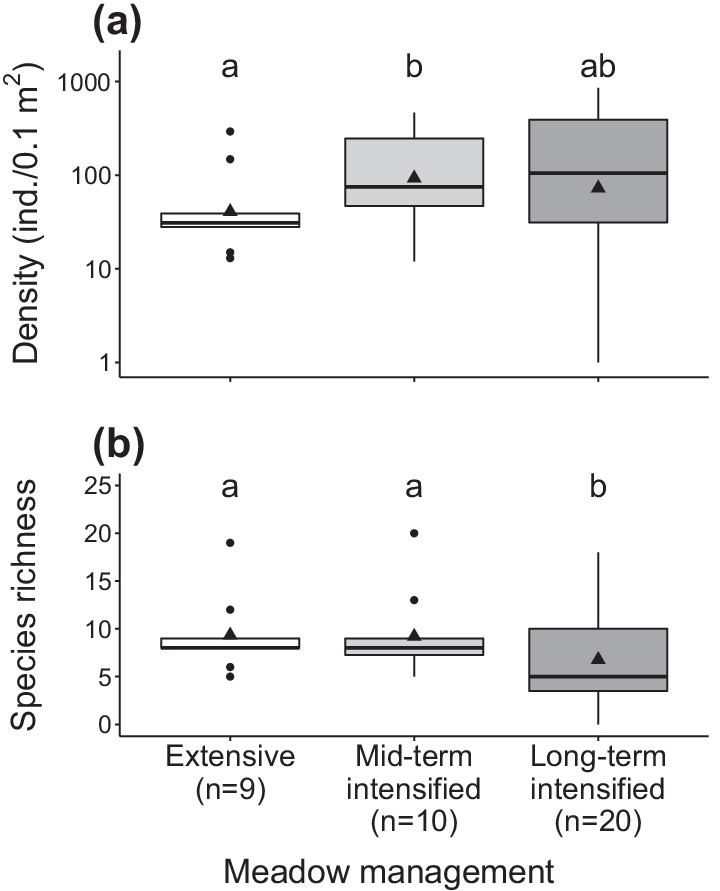
Effects of meadow management intensification on **a** snail density and **b** species richness. Data for the extensive (no water and fertiliser inputs, i.e. control plots) and mid-term intensive (plots having received high inputs of water and fertiliser during 5 years) management types stemmed from the experimental module, whereas data from the long-term intensive management (> 20 years) are drawn from the observational module. Bold lines represent box-plot medians, solid triangles means, boxes the first and third quantiles, whiskers the inter-quartile distance multiplied by 1.5, and solid dots the outliers. Note the log-scale on the *y*-axis in graph (**a**). Different letters indicate significant differences between treatments at *P* < 0.05

Snail densities in long-term intensively-managed meadows (mean ± standard error = 43.2 ± 18.2 per 0.1 m^2^) lied between those in extensive (37.6 ± 17.5) and mid-term intensified plots (93.8 ± 42.3; Fig. 1a). Species richness in long-term intensively-managed meadows was about 35% lower (5.4 ± 0.8) than in extensive (8.1 ± 1.3) and mid-term intensified plots (8.3 ± 1.3, Fig. 1b). Similarly, evenness was significantly lower in long-term intensively-managed (0.75 ± 0.02) than in extensively-managed plots (0.88 ± 0.03), and similar compared to mid-term intensified plots (0.82 ± 0.03, *P* = 0.088; Additional file 4: Appendix S4).

**Fig. 2:**
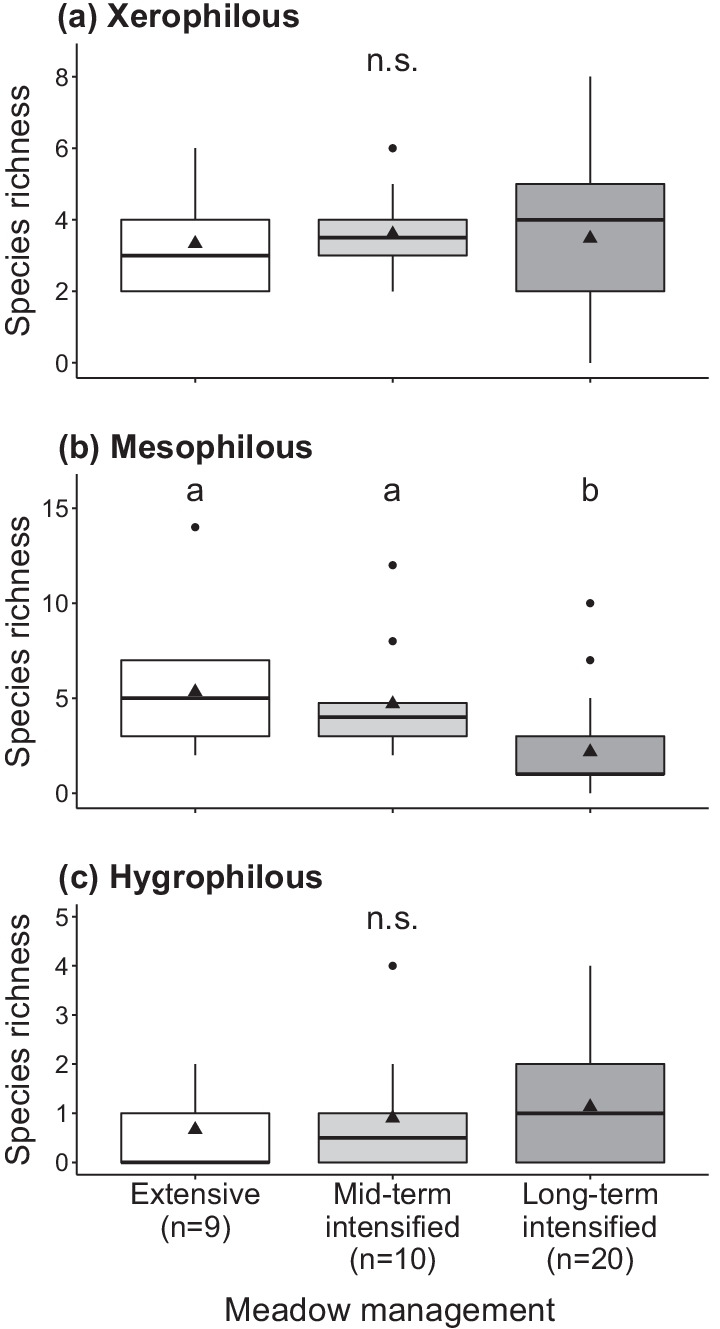
Effect of meadow management intensification on species richness of land snails split according to their moisture preference: **a** xerophilous; **b** mesophilous; **c** hygrophilous. For management descriptions and box-plot features, see legend of Fig. 1

**Fig. 3:**
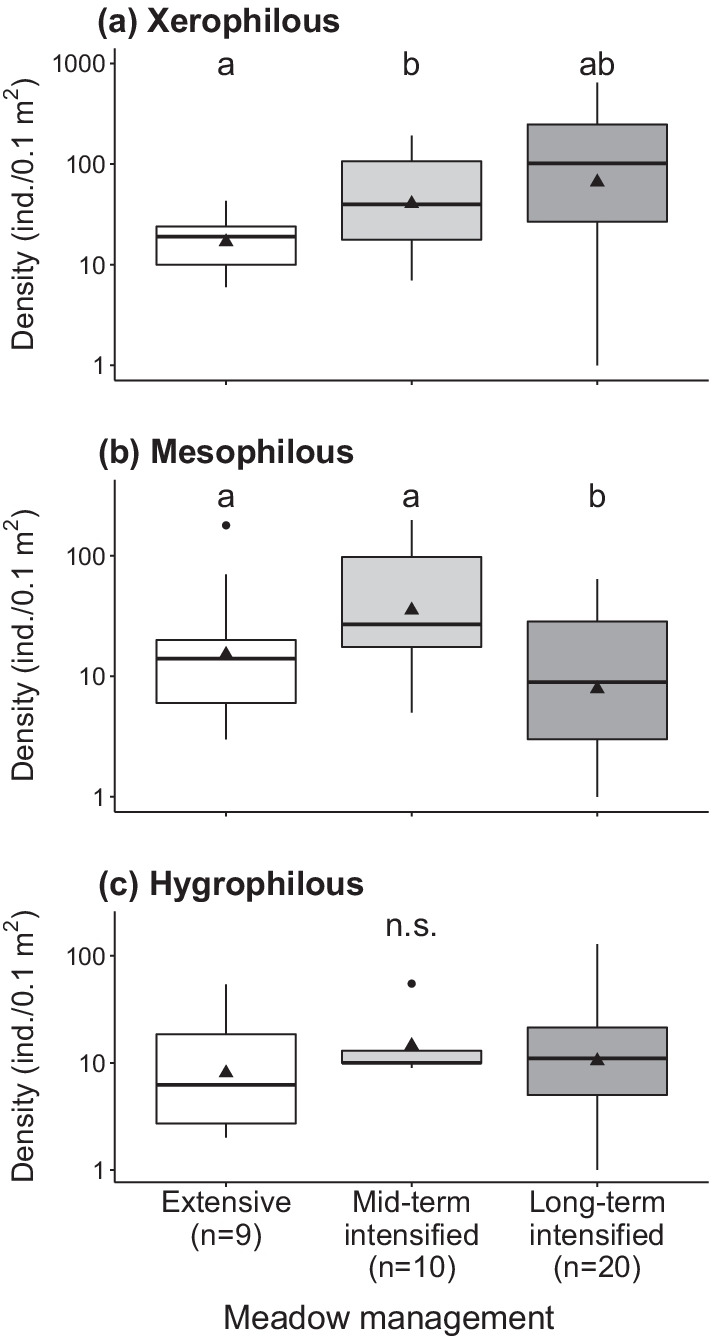
Effect of meadow management intensification on densities of land snails split according to their moisture preference: **a** xerophilous, **b** mesophilous, **c** hygrophilous. Note the log-scale on the *y*-axis of the graphs. For management descriptions and box-plot features, see legend of Fig. 1

Analyses of snail density and richness according to their soil moisture preferences showed that the richness of xerophilous species was similar in all management types (Fig. 2a), but their density was significantly lower in extensively-managed plots (14.4 ± 6.9) than in the other management types (mid-term intensified: 37.3 ± 17.2; long-term intensified: 32.7 ± 13.8; Fig. 3a, Additional file 4: Appendix S4). Moreover, the CWM of moisture preferences was lowest (i.e. greater contribution of drought-tolerant species) in long-term intensively-managed meadows (Additional file 4: Appendix S4). Long-term intensive management affected mesophilous snail species, with ~ 60% fewer species (1.6 ± 0.4) than in mid-term intensified (3.8 ± 0.8) and in extensively-managed plots (4.1 ± 0.9, Fig. 2b). Likewise, densities of mesophilous snails in long-term intensively-managed meadows (3.5 ± 1.5) were lower than in mid-term intensified (37.3 ± 16.9) and extensively-managed plots (15.5 ± 7.38; Fig. 3b). Hygrophilous species were similarly scarce in all treatments (Fig. 2c). No significant differences were found for the density of hygrophilous snails nor for the number of red-listed species (Fig. 3c; Additional file 4: Appendix S4).

Community analysis was performed with 13 species (*Candidula unifasciata*, *Ceciliodes acicula*, *Cochlicopa lubrica*, *C. lubricella*, *Punctum pygmaeum*, *Pupilla muscorum*, *Trochulus* sp., *Truncatellina cylindrica*, *Vallonia costata*, *V. excentrica*, *V. pulchella*, *Vertigo pygmaea* and *Xerolenta obvia*) occurring in at least nine samples across both modules. This analysis revealed that community composition differed with time exposure to grassland intensification (Additional file 4: Appendix S4). The species that contributed most to these differences were *Pupilla muscorum* (more abundant in mid-term intensified plots and long-term intensively-managed meadows, *P* = 0.056), *Cochlicopa lubricella* and *Punctum pygmaeum* (both more abundant in extensively-managed and mid-term intensified plots, *P* = 0.029 and *P* = 0.026, respectively). Community composition differed as well between study sites. For detailed model outputs and graphs, see Additional file 4: Appendix S4.

### Mid-term effects of grassland management intensification on land snail communities (experimental module)

**Fig. 4:**
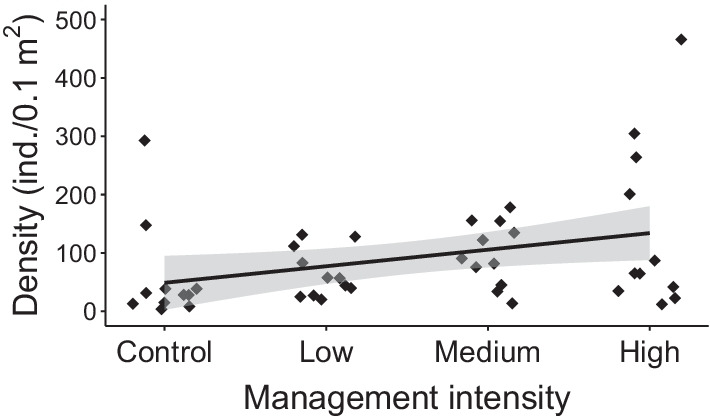
Snail density in response to the management intensity gradient in the experimental module, consisting of control (no inputs), low-, medium- and high-input levels of water and fertiliser. The black line represents the fitted model, along with a 95% confidence band in grey

Snail densities (Fig. 4) and soil pH increased along the management intensification gradient, whereas evenness declined slightly (Additional file 5: Appendix S5). Furthermore, densities of both xerophilous and mesophilous snails were positively influenced by mid-term management intensification. Significant effects were found neither for overall species richness nor for moisture preference groups (Additional file 5: Appendix S5). Hygrophilous species were scarce at all study sites, so that it was not possible to use this group in the analysis.

Community analysis was performed with 14 species (*Ceciliodes acicula*, *Cochlicopa lubricella*, *Nesovitrea hammonis*, *Punctum pygmaeum*, *Pupilla muscorum*, *Succinella oblonga*, *Trochulus* sp., *Truncatellina cylindrica*, *Vallonia costata*, *V. excentrica*, *V. pulchella*, *Vertigo pygmaea* and *Vitrina pellucida*) occurring in at least nine plots of the experimental module. Most species contributed to a differentiation of the community composition with the intensification gradient, but only *Vallonia costata* showed a marginally significant univariate positive response (*P* = 0.058; Additional file 5: Appendix S5). Community composition differed as well between study sites. For detailed model outputs and graphs, see Additional file 5: Appendix S5.

### Environmental variables influencing land snail community composition in long-term intensively-managed meadows (observational module)

**Fig. 5:**
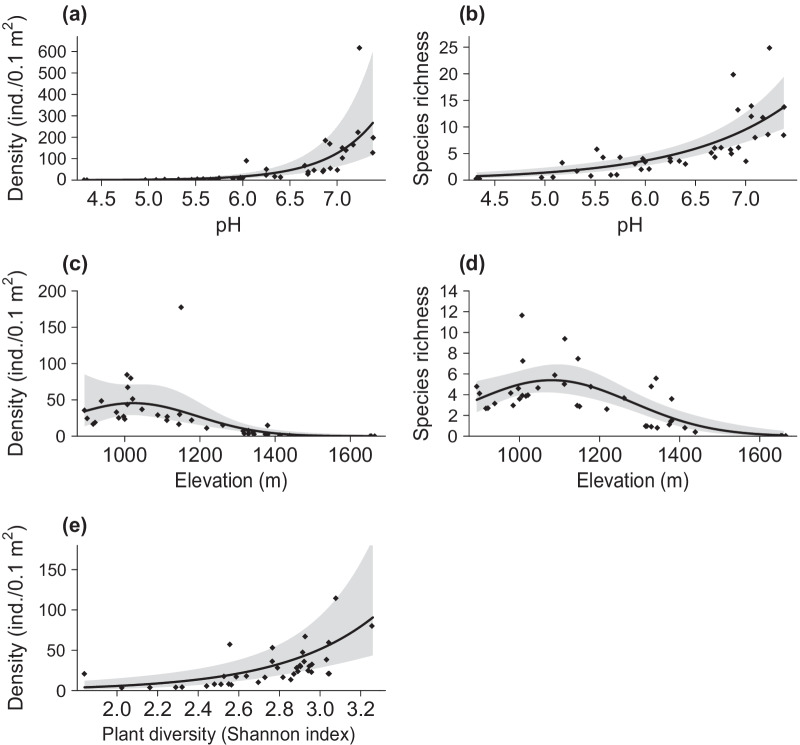
Effect plots with partial residuals (i.e. the residuals left after subtracting the influence of the other variables in the model) for each of the predictors influencing snail density and snail species richness: **a**, **b** soil pH; **c**, **d** elevation; and **e** plant diversity (observational module). The black line in each plot represents the fitted model with a 95% confidence band (grey area)

Soil pH stood out as the most important variable in the study system, having the highest influence on snail density, richness and the number of red-listed species in a positive manner (Fig. 5a, b and Additional file 6: Appendix S6). Elevation had a quadratic effect with an optimum at around 1100 m a.s.l. (Fig. 5c, d), but its influence on snail density and species richness was generally negative. Finally, plant diversity (Shannon index) significantly enhanced snail density (Fig. 5e). For detailed model outputs and graphs, see Additional file 6: Appendix S6.

## Discussion

Mountain grassland management intensification, through inputs of fertiliser in the form of slurry and irrigation with sprinklers, imposes novel microhabitat conditions that compromise both plant and invertebrate biodiversity (e.g. [13]). However, the impact of such practices on land snails remained poorly-understood until the present study. Given their low mobility and affinity for moisture, it was suspected that mid- and long-term exposure of land snail communities to agricultural intensification would show a progressive impoverishment of species assemblages but not necessarily a decrease in abundance. Our study confirms that community composition changed over time. Although snail densities were promoted in the mid-term following management intensification (within 5 years), they ended up with lower densities and ~ 35% fewer species in the long-term (> 20 years). Contrary to our expectations, however, drought-tolerant (i.e. xerophilous) species were predominant and even more abundant in intensively-managed meadows in the long term, while mesophilous species seemed to lose ground. We shall next discuss more in detail the effects of management intensification on land snails during the course of time, focus on the mid-term responses observed in the experimental module and finally examine the key environmental variables shaping snail communities in long-term intensively-managed mountain meadows (observational module), before concluding with management recommendations.

### Land snail communities are particularly affected by intensification in the long run

By comparing snail assemblages of mountain hay meadows that were either extensively-managed, intensified for 5 years (extensively-managed beforehand) or intensively-managed for at least 20 years, we could demonstrate that snail communities get impoverished under a long-term intensification regime. Mesophilous species, which typically occur in either dry or moist environments, were particularly impacted: they were underrepresented (minus ~ 60% species richness) in long-term intensively-managed meadows compared to the other management types. We had predicted that mesophilous species would benefit from the more shaded and wetter conditions prevailing at ground level due to a denser vegetation sward generated by intensification [13, 23, 47], but found a different pattern. A posteriori, we interpret these unexpected results, first, by an increased mowing frequency in intensively-managed meadows, which impacts mesophilous species more severely than xerophilous species [22, 48]. In effect, under these circumstances, the former species, generally more sensitive to desiccation, had to endure more frequent periods with no vegetation cover, i.e. longer exposure to direct solar radiation, with detrimental effects on their populations [49]. In contrast, xerophilous species have a greater natural ability to cope with xeric circumstances [50, 51] and were thus particularly well represented in intensively-managed meadows. This is well illustrated by the species *Pupilla muscorum*, that was most abundant in long-term intensively-managed meadows. Interestingly, this species characteristic of open-land habitat was established to vanish from Alpine hay meadows after agricultural abandonment [47], which corroborates our interpretation. Second, direct mortality caused by mowing machinery might also account for community impoverishment in long-term intensively-managed meadows [37, 52, 53]. However, a majority of the species recorded are ground-dwellers [23] so that this mortality source must be considerably lower than the mortality elicited by habitat alterations [52–54]. Third, a mere sampling year effect may in theory explain some of the differences observed between snail communities in the mid- and long-term [22, 32]. Yet, the fact that snail abundance was not affected while species richness was, argues against this interpretation. Finally, the decline in plant diversity driven by management intensification in the long-term [55, 56] could contribute to level off the mid-term positive response of snail density that follows intensification, probably due to overall ecological niche space reduction (see subsection ‘Key environmental factors shaping snail communities in mountain hay meadows’).

Our results for hay meadows differ markedly from findings obtained in pastures where intensive management via fertilizer application and grazing seems to negatively impact both snail density and species richness, especially of xerophilous species [57]. This difference is probably due to the negative effects of cattle trampling on soil fauna that increase with increased grazing pressure [58]. Furthermore, our meadows were not harbouring the very specialised xerophilous species of conservation concern that typically inhabit the dry steppic slopes of Valais [37], which is probably due to an absence of key structural elements such as rocks and wide patches of bare ground [37, 57].

### Mid-term benefits of intensification for land snail communities

In our controlled experiment, the intensification of grassland management through site-adapted irrigation and fertilisation had boosted land snail densities after 5 years, this without compromising species richness. The highest snail densities (2.7 greater than in the extensively-managed, control plots) were reached when irrigation and fertilisation were combined at the levels needed to achieve maximum local hay yield (see ‘Methods’). Since similar results had been reported for sward-dwelling snails in the same study meadows [32], we can generalise these effects to the entire meadow snail community [59]. Again, it is likely that the wetter and cooler microclimate generated by a denser vegetation favoured snails [13, 27, 60], providing them with better conditions for oviposition and egg survival, thus boosting their population sizes [61]. This interpretation of a primary effect of microclimate, instead of overall increased phytomass, is further supported by the recognition that food supply is generally not a limiting factor for snail populations [62, 63]. Increased nitrogen availability following fertilisation [64] can also not be inferred in the present case: the plants in our fertilised plots did not have higher nitrogen content than in control plots [65].

Remarkably, mesophilous snails were unaffected by mid-term intensification. On the contrary, they had even augmented in numbers after 5 years of experimental intensification, but this state was only transient as they showed a marked decline in the long run (see ‘Land snail communities are particularly affected by intensification in the long run’).

Our results also show that grassland farming intensification causes an increment of soil pH, most likely due to the buffering action of the organic compounds contained in organic fertilisers such as slurry [66]. A resulting lower acidity among intensively-managed meadows apparently benefit snail communities, particularly those adapted to moderately acid to neutral soils (see also ‘Key environmental factors shaping snail communities in mountain hay meadows’).

### Key environmental factors shaping snail communities in mountain hay meadows

Soil pH, plant diversity and elevation were all identified as key environmental factors shaping the communities of land snails inhabiting those of our mountain meadows that had been intensively managed for at least the previous 20 years. Almost no snails were found in meadows with soil pH < 5.5, corroborating former findings in various habitats (e.g. [30, 54, 67]). The mechanism at play is evident: snails need access to sources of calcium, in particular for building their shells [61, 68], but this mineral is common only in soils on a limestone substrate, and rare in soils on silicate substrates (granite, gneiss).

The positive effect of plant diversity on land snails, essentially on their density, was remarkable but not novel [62, 69]. Albeit the underlying mechanism remains ill-understood, it is likely that a diverse plant community enhances the microhabitat structural complexity [69] that is necessary at different stages of a snail’s life cycle (oviposition site, shelter, etc.) [27].

Lastly, the negative effect of elevation on our grassland snail communities is in line with the findings by Schmera and Baur [70] who reported a decrease of gastropod abundance along an elevational gradient. This is explained by the fact that the activity period of land snails depends on the length of the growing season, which shortens towards higher elevations. Note that the modest peak of abundance and richness we observed at ca 1′100 m a.s.l. reflects a spatial clustering of our most specious meadows at that elevation. This hump-shaped vertical distribution may be due to even more intensive farming practices in grasslands next to the plain (400–550 m a.s.l.) have led to extremely impoverished snail communities in the long run.

## Conclusions

A major, and unexpected finding of this study is that drought-tolerant snail species remain fairly unharmed and even proliferate with grassland intensification in mountain hay meadows, this despite the fact that snail communities get altogether impoverished under the pressure of intensification in the long run. Certainly, the present results and management recommendations do not readily apply to lowland meadows where levels of intensification are of another order of magnitude, creating conditions way more hostile for biodiversity. If intensification of mountain hay meadows provides short-term benefits for snail communities via enhanced moisture and shade at ground level, it eliminates in the long run the most sensitive species to the very dry environmental conditions that characterise the post-mowing period. This phenomenon is exacerbated by the limited active mobility of snails [22, 71, 72] that represents a natural impediment to any recolonization process from nearby species reservoirs [30, 73, 74]. This calls for conserving in priority meadows with a high land snail diversity, as well as promoting uncut refuge strips to preserve areas without disturbances related to mowing [24]. Finally, if land snails are certainly not the best candidates to serve as bioindicators of integral meadowland invertebrate communities, we think they deserve more attention from conservation and restoration programmes aiming to preserve the whole set of interactions and functions that characterise biodiversity-rich montane and subalpine grassland ecosystems.

## Supplementary Information

**Additional file 1: Appendix S1.** Description the study sites.

**Additional file 2: Appendix S2.** Explanatory variables of the observational module.

**Additional file 3: Appendix S3.** Snail species list.

**Additional file 4: Appendix S4.** Results of the response of snail communities to long-term management intensification (both modules).

**Additional file 5: Appendix S5.** Results of the experimental module.

**Additional file 6: Appendix S6.** Results of the observational module.

## Data Availability

The R script and complete dataset supporting the conclusions of this article are available in the Figshare repository: https://doi.org/10.6084/m9.figshare.12957776.v4. Complementary results and figures as well as model outputs are provided as Additional files 1, 2, 3, 4, 5, and 6.

## Abbreviation

CWM: Community weighted mean
