# Supplementary material for: Mid- and long-term responses of land snail communities to the intensification of mountain hay meadows management
Source: BMC Ecol Evol. 2022 Feb 15;22:19. doi: 10.1186/s12862-022-01972-4 (PMC8845342; doi:10.1186/s12862-022-01972-4)
Supplement: Supplementary file 1 — Additional file 1: Appendix S1. Description the study sites. [file 12862_2022_1972_MOESM1_ESM.docx]

**Mid- and long-term responses of land snail communities to the intensification of mountain hay meadows management**

Gerard Martínez-De León^a, *^, Lauriane Dani^a^, Aline Hayoz-Andrey^a^, Ségolène Humann-Guilleminot^a^, Raphaël Arlettaz^a^ and Jean-Yves Humbert^a^

^a^ Division of Conservation Biology, Institute of Ecology and Evolution, University of Bern, Baltzerstrasse 6, 3012 Bern, Switzerland

*Corresponding author

Email: [gerard.martinezdeleon@iee.unibe.ch](mailto:gerard.martinezdeleon@iee.unibe.ch); [martinezdeleongerard@gmail.com](mailto:martinezdeleongerard@gmail.com)

**Appendix S1 – Description of the study sites**

This appendix provides the location and soil pH of the study sites in both experimental and observational modules, along with a description of the management treatments of the experimental module.

Table of content

| Fig. S1.1 | Topographical map displaying the location of the study sites in the canton of Valais, Inner Swiss Alps |
| --- | --- |
| Table S1.1 | Description of each meadow of the experimental module |
| Table S1.2 | Management treatments applied on each meadow of the experimental module |
| Table S1.3 | Description of each meadow of the observational module |


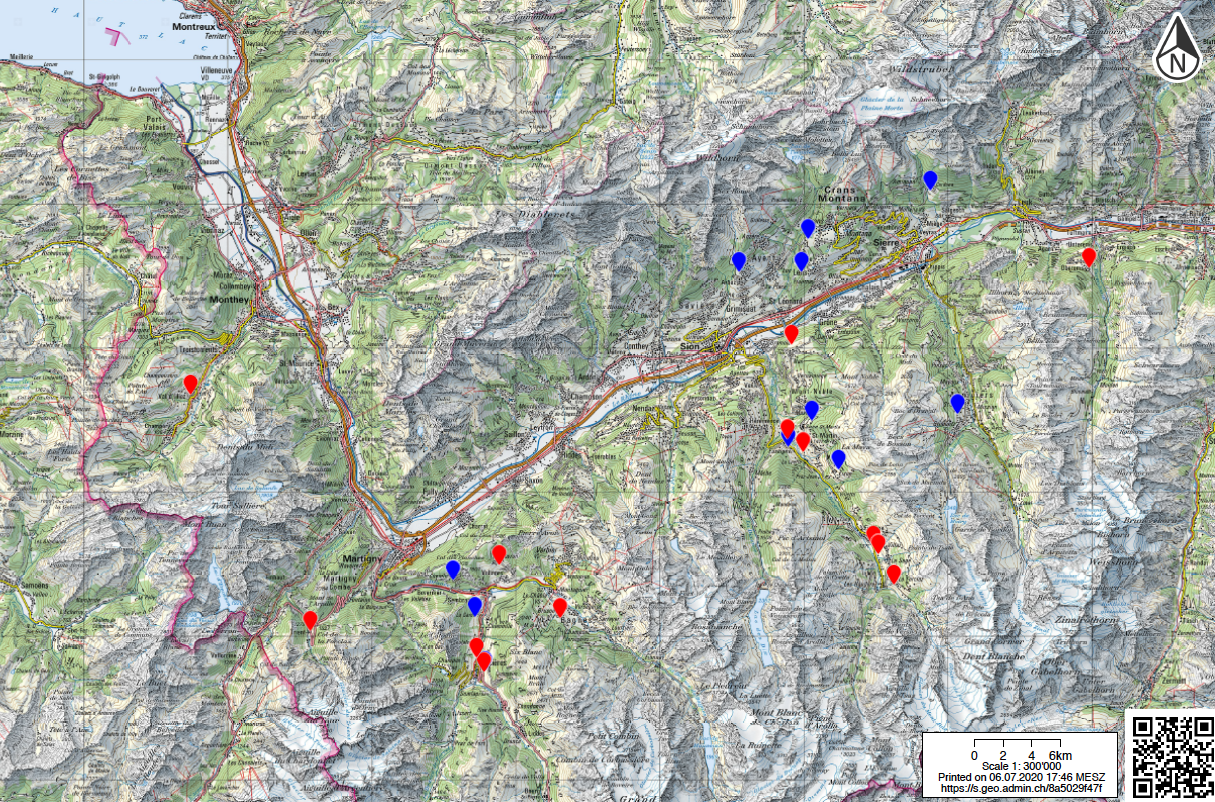


**Fig. S1.1.** Topographical map (1:300000) displaying the locations of the study sites in the canton of Valais, Inner Swiss Alps. The climate in the region is continental, with a mean annual precipitation of 603 mm and monthly average temperatures ranging from -0.1 °C in January, to 20.1 °C in July, recorded at the valley bottom in Sion (482 m.a.s.l.) between 1981 and 2010 (MeteoSwiss, 2019). Blue markers represent the locations of the extensively managed meadows of the experimental module. Red markers show the locations of the thirteen regions of the observational module, with three long-term intensively managed meadows selected per region (39 meadows in total). Source: Federal Office of Topograhpy swisstopo.

**Table S1.1**. Description of each meadow of the experimental module, including the name of the study site, elevation, and coordinates (WGS 84). Soil pH is used to define the subset of samples above a threshold of pH 6 in the analyses on the response of land snails to long-term grassland intensification. Therefore. the values of soil pH are provided for the extensive and recently intensified plots, and those included in the analyses are marked with an asterisk (*).

| Study site | Elevation [m] | Soil pH | |  | Coordinates | |
| --- | --- | --- | --- | --- | --- | --- |
|  |  | C-plots | I+F 3/3-plots |  | Latitude | Longitude |
| Icogne 1 | 1200 | 6.0 | 7.0* |  | 46°17′56″N | 7°26′31″E |
| Icogne 2 | 880 | 7.4* | 7.6* |  | 46°16′42″N | 7°26′10″E |
| La Garde | 880 | 7.4* | 7.6* |  | 46°16′42″N | 7°26′10″E |
| Vens | 1373 | 6.4* | 7.3* |  | 46°5′7″N | 7°7′24″E |
| Arbaz | 980 | 6.4* | 7.0* |  | 46°3′45″N | 7°8′35″E |
| Cordona | 1153 | 6.9* | 7.1* |  | 46°19′45″N | 7°33′8″E |
| Eison | 1373 | 6.5* | 6.8* |  | 46°5′7″N | 7°7′24″E |
| Saint-Martin | 1589 | 6.1* | 6.4* |  | 46°11′8″N | 7°26′43″E |
| Grimentz | 1270 | 5.5 | 5.7 |  | 46°16′42″N | 7°22′47″E |
| Orsières 1 | 1022 | 7.6* | 7.7* |  | 46°1’ 44”N | 7° 9’ 8”E |
| Euseigne | 1028 | 7.4* | 7.4* |  | 46° 10’ 9”N | 7° 25’ 27”E |

**Table S1.2.** Management treatments applied on each meadow of the experimental module. These treatments consisted of control (no input), low-, medium- and high-input levels of fertilizer and irrigation, mimicking a management intensification gradient. Note that all meadows were managed extensively (i.e. any or minor application of fertiliser and irrigation) before the onset of the experiment. For each treatment indications are provided for: quantity of nitrogen (N), phosphorus (P) and potassium (K) fertiliser applied per hectare and year; amount of irrigation applied per week via sprinkler; and number of grass cuts per year. The fertiliser consisted of organic NPK pellets and mineral K_2_O dissolved in water to reach the equivalent of standard-farm liquid manure. The amount of NPK depended on the potential productivity of each meadow, within the given range of values (for further details on the experimental design see Appendix A in Andrey, Humbert, & Arlettaz, 2016).

| Management treatments | Number of cuts per year | Fertiliser applied [kg·ha^−1^·year^−1^] | | | Water irrigation [mm·week^−1^] |
| --- | --- | --- | --- | --- | --- |
|  |  | N | P | K |  |
| Control | 1 | 0 | 0 | 0 | 0 |
| Low | 2 | 13.3 – 26.7 | 4.8 – 9.7 | 36.9 – 73.8 | 10 |
| Medium | 2 | 26.7 – 53.3 | 9.6 – 19.4 | 73.8 – 147.5 | 20 |
| High | 2 | 40 - 80 | 14.5 – 29.1 | 110.6 – 221.4 | 30 |

**Table S1.3**. Description of each meadow of the observational module, including the name of the study site, elevation, soil pH, and coordinates (WGS 84). Note that each study site contained three long-term intensively managed meadows in close proximity. The sites above a threshold of pH 6 are marked with an asterisk (*), as they were included in the analyses on the response of land snails to long-term grassland intensification.

| Study site | Elevation [m] | Soil pH | Coordinates | |
| --- | --- | --- | --- | --- |
|  |  |  | Latitude | Longitude |
| Bruson | 1112 | 5.9 | 46° 3’ 43”N | 7° 13’ 10”E |
|  | 1113 | 5.6 | 46° 3’ 43”N | 7° 13’ 8”E |
|  | 1088 | 6.0 | 46° 3’ 35”N | 7° 13’ 23”E |
| Orsières 1 | 1008 | 6.9* | 46° 1’ 41”N | 7° 9’ 5”E |
|  | 1006 | 6.9* | 46° 1’ 37”N | 7° 9’ 5”E |
|  | 1007 | 7.1* | 46° 1’ 39”N | 7° 9’ 5”E |
| Orsières 2 | 938 | 7.2* | 46° 2’ 12”N | 7° 8’ 35”E |
|  | 900 | 7.1* | 46° 2’ 14”N | 7° 8’ 41”E |
|  | 893 | 7.1* | 46° 2’ 9”N | 7° 8’ 42”E |
| Val d’Illiez | 1000 | 5.3 | 46° 12’ 1”N | 6° 53’ 11”E |
|  | 978 | 6.3* | 46° 11’ 59”N | 6° 53’ 13”E |
|  | 997 | 6.0 | 46° 12’ 2”N | 6° 53’ 13”E |
| Le Levron | 1178 | 7.4* | 46° 5’ 42”N | 7° 10’ 6”E |
|  | 1218 | 7.4* | 46° 5’ 42”N | 7° 9’ 53”E |
|  | 1261 | 7.2* | 46° 5’ 49”N | 7° 9’ 53”E |
| Nax | 1150 | 6.0 | 46° 13’ 59”N | 7° 25’ 43”E |
|  | 1144 | 6.0 | 46° 13’ 59”N | 7° 25’ 38”E |
|  | 1146 | 5.8 | 46° 13’ 59”N | 7° 25’ 40”E |
| La Luette | 1021 | 6.6* | 46° 9’ 56”N | 7° 26’ 16”E |
|  | 1016 | 6.3* | 46° 9’ 57”N | 7° 26’ 15”E |
|  | 984 | 6.7* | 46° 9’ 58”N | 7° 26’ 11”E |
| Euseigne | 1046 | 6.3* | 46° 10’ 16”N | 7° 25’ 6”E |
|  | 916 | 6.9* | 46° 10’ 26”N | 7° 25’ 25”E |
|  | 921 | 6.9* | 46° 10’ 25”N | 7° 25’ 30”E |
| Evolène | 1374 | 6.9* | 46° 6’ 26”N | 7° 30’ 2”E |
|  | 1378 | 6.8* | 46° 6’ 27”N | 7° 30’ 2”E |
|  | 1380 | 7.2* | 46° 6’ 36”N | 7° 29’ 31”E |
| La Tour | 1380 | 6.4* | 46° 6’ 9”N | 7° 30’ 5”E |
|  | 1413 | 6.7* | 46° 6’ 7”N | 7° 30’ 18”E |
|  | 1439 | 7* | 46° 6’ 11”N | 7° 30’ 23”E |
| La Forclaz | 1656 | 5.7 | 46° 4’ 59” N | 7° 31’ 8”E |
|  | 1665 | 5.5 | 46° 5’ 24”N | 7° 30’ 54”E |
|  | 1653 | 5.7 | 46° 5’ 27”N | 7° 30’ 54”E |
| Trient | 1318 | 4.4 | 46° 3’ 10”N | 6° 59’ 44”E |
|  | 1315 | 4.3 | 46° 3’ 12”N | 6° 59’ 44”E |
|  | 1329 | 5.0 | 46° 3’ 6”N | 6° 59’ 46”E |
| Oberems | 1341 | 5.5 | 46° 16’ 58”N | 7° 41’ 11”E |
|  | 1344 | 5.1 | 46° 16’ 50”N | 7° 41’ 42”E |
|  | 1329 | 5.2 | 46° 16’ 59”N | 7° 41’ 9”E |

**References**

Andrey, A., Humbert, J.-Y., & Arlettaz, R. (2016). Functional response of leaf- and planthoppers to modern fertilisation and irrigation of hay meadows. *Basic and Applied Ecology*, *17*(7), 627–637. https://doi.org/10.1016/j.baae.2016.07.002

MeteoSwiss, F. O. of M. and C. (2019). Normals 1981-2010: Precipitation total and air temperature. *Swiss Confederation*, 1–9. Retrieved from http://www.meteoswiss.admin.ch/files/kd/normwerte/norm8110/nvrep_tre200m0_en.pdf
