# Supplementary material for: Mid- and long-term responses of land snail communities to the intensification of mountain hay meadows management
Source: BMC Ecol Evol. 2022 Feb 15;22:19. doi: 10.1186/s12862-022-01972-4 (PMC8845342; doi:10.1186/s12862-022-01972-4)
Supplement: Supplementary file 2 — Additional file 2: Appendix S2. Explanatory variables of the observational module. [file 12862_2022_1972_MOESM2_ESM.docx]

**Mid- and long-term responses of land snail communities to the intensification of mountain hay meadows management**

Gerard Martínez-De León^a, *^, Lauriane Dani^a^, Aline Hayoz-Andrey^a^, Ségolène Humann-Guilleminot^a^, Raphaël Arlettaz^a^ and Jean-Yves Humbert^a^

^a^ Division of Conservation Biology, Institute of Ecology and Evolution, University of Bern, Baltzerstrasse 6, 3012 Bern, Switzerland

*Corresponding author

Email: [gerard.martinezdeleon@iee.unibe.ch](mailto:gerard.martinezdeleon@iee.unibe.ch); [martinezdeleongerard@gmail.com](mailto:martinezdeleongerard@gmail.com)

**Appendix S2** - **Explanatory variables of the observational module**

**Table S2.1.** Description of all explanatory variables collected in the observational module. According to their properties, variables were grouped into topographical, soil, vegetation and landscape related type of variables.

| Type of variable | Variable | Definition |
| --- | --- | --- |
| Topographical | Elevation | Meters above sea level |
|  | Slope | Steepness of the meadow [°], measured using a compass with clinometer |
|  | Folded aspect | Exposition of the meadow [°], measured using a compass with clinometer. The values were later rescaled into 0-180° following McCune & Dylan (2002), resulting into a proxy of potential direct incident radiation |
|  | Heat load | Index based on latitude, slope and aspect of the sites. Higher values of this index indicate more heat received in the meadow by incident radiation (McCune & Dylan, 2002) |
| Soil | pH | Acidity of the soil (acid: [1-6], neutral: [7] and basic: [8-14]) |
|  | Grain size distribution | Proportion of clay (0.02 μm - 2.00 μm), silt (2.00 – 63.00 μm) and sand (63.00 – 2000.00 μm) |
|  | Inorganic and total carbon | Carbon concentration in the soil [% of weight]. Inorganic carbon was measured as the difference between total carbon and organic carbon after reaction with HCl |
|  | Nitrogen | Nitrogen concentration in the soil [% of weight] |
|  | C:N ratio | Ratio of carbon and nitrogen in the soil, as a measure of nitrogen available for plant uptake (Hodge, Robinson, & Fitter, 2000) |
| Vegetation | Plant species richness | Total number of species recorded in the two vegetation plots |
|  | Shannon index of plant diversity | Plant species richness weighted with the percentage cover |
|  | Landolt humidity - Community Weighted Mean | Value of each plant species according to their soil humidity requirements, from 1 (very dry) to 5 (aquatic), weighted by their cover in the plot (Landolt et al., 2010) |
|  | Cover of plant functional groups (forbs, grasses, legumes) | Groups were defined according to the family to which each plant species belonged: legumes (Fabaceae), grasses (Poaceae, Juncaceae, Cyperaceae) and forbs (other families). |
| Type of variable | Variable | Definition |
| Vegetation | Bare ground | Visual estimation of the cover of bare ground [%] |
|  | Litter | Visual estimation of the cover of litter [%] |
|  | Mean vegetation height | Mean height [cm] of every contact point with a plant in measuring location. This was done in 10 locations along two diagonal transects crossing the entire meadow, once before each hay cut. |
| Management | Irrigation | Presence or absence of irrigation with sprinklers |
| Landscape | Grassland | Relative cover of semi-natural grasslands (i.e. meadows and pastures) in a 50 m buffer around the study meadows [%] |
|  | Forest | Relative cover of forests (i.e. coniferous, mixed, broadleaved) in a 50 m buffer around the study meadows [%] |
|  | Extensive semi-natural structures | Relative cover of extensively managed structures (i.e. grasslands with low productivity, steppe-like vegetation) in a 50 m buffer around the study meadows [%] |
|  | Artificial structures | Relative cover of artificial structures (i.e. buildings, paved roads) in a 50 m buffer around the study meadows [%] |

**
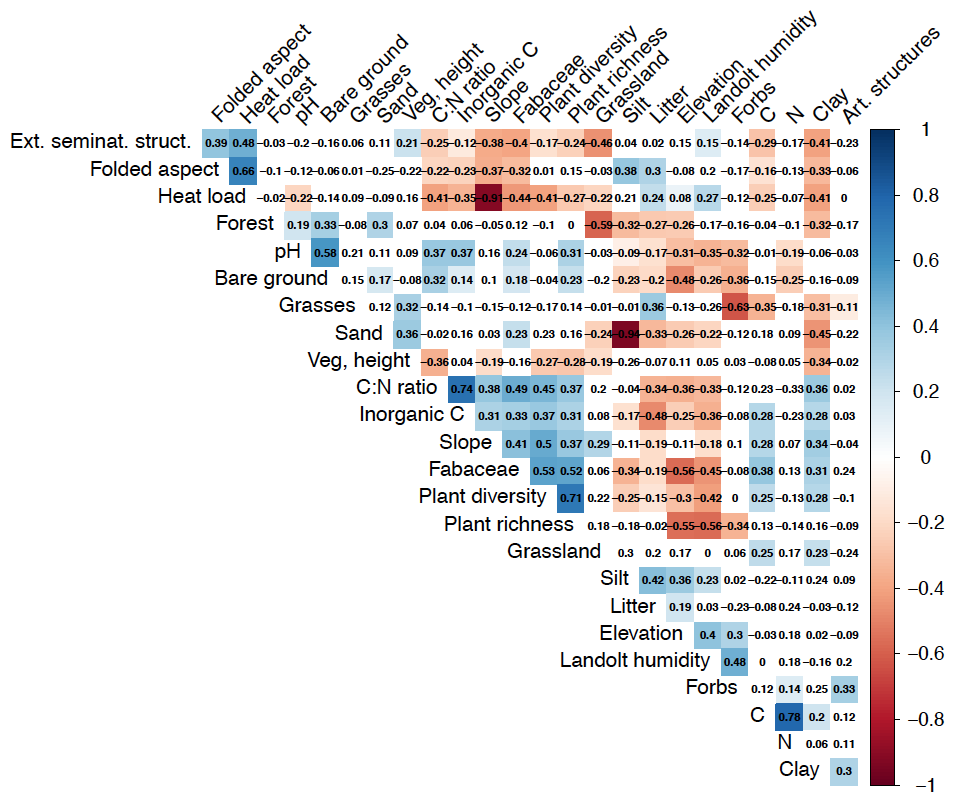
**

**Figure S2.1.** Correlation plot of all continuous variables with Spearman correlation values. Significant correlations (*P* < 0.05) have a coloured background in blue (positive correlation) or red (negative correlation). After assessing the variables having a pairwise correlation coefficient > 0.7, the following variables were removed from the analyses: *inorganic C*, *N content*, *plant richness, sand, silt* and *slope*. *Silt* and *sand* could be merged into a single variable, but instead we decided to use the variable *clay,* as it represents the complementary proportion. *Folded aspect* was also removed because it is involved in the calculation of *heat load* and it is considerably correlated with this variable (ρ = 0.66).

**References**

Hodge, A., Robinson, D., & Fitter, A. (2000). Are microorganisms more effective than plants at competing for nitrogen? *Trends in Plant Science*, *5*(7), 304–308.

Landolt, E., Bäumler, B., Erhardt, A., Hegg, O., Klötzli, F., Lämmler, W., … Wohlgemuth, T. (2010). *Flora indicativa. Ökologische Zeigerwerte und biologische Kennzeichen zur Flora der Schweiz und der Alpen. Ecological indicators values and biological attributes of the flora of Switzerland and the Alps (2nd ed.)* (2nd ed.). Haupt.

McCune, B., & Dylan, K. (2002). Equations for potential annual direct incident radiation and heat load. *Journal of Vegetation Science*, *13*, 603–606.
