# Supplementary material for: Mid- and long-term responses of land snail communities to the intensification of mountain hay meadows management
Source: BMC Ecol Evol. 2022 Feb 15;22:19. doi: 10.1186/s12862-022-01972-4 (PMC8845342; doi:10.1186/s12862-022-01972-4)
Supplement: Supplementary file 3 — Additional file 3: Appendix S3. Snail species list. [file 12862_2022_1972_MOESM3_ESM.docx]

**Mid- and long-term responses of land snail communities to the intensification of mountain hay meadows management**

Gerard Martínez-De León^a, *^, Lauriane Dani^a^, Aline Hayoz-Andrey^a^, Ségolène Humann-Guilleminot^a^, Raphaël Arlettaz^a^ and Jean-Yves Humbert^a^

^a^ Division of Conservation Biology, Institute of Ecology and Evolution, University of Bern, Baltzerstrasse 6, 3012 Bern, Switzerland

*Corresponding author

Email: [gerard.martinezdeleon@iee.unibe.ch](mailto:gerard.martinezdeleon@iee.unibe.ch); [martinezdeleongerard@gmail.com](mailto:martinezdeleongerard@gmail.com)

**Appendix S3 – Snail species list**

**Table S3.1**. List of snail species and absolute abundance of fresh shells across all study sites. Nomenclature followed Hausser (2005), with consideration for later taxonomical updates (Horsáková, Nekola, & Horsák, 2020). All the individuals were identified to species level, except the immature shells of *Cochlicopa*, *Vallonia* and *Vertigo* when different species belonging to the same genus could be found in a sample. In this case, it was not possible to allocate non-fully developed shells to a particular species with confidence. Furthermore, individuals of the genus *Trochulus* could not be identified to species level, as the two species to which they belonged (*T. hispida* and *T. sericeus*) are hardly distinguishable. All two-toothed individuals of *Pupilla* were considered as *Pupilla muscorum*, given that identifications based on apertural features in *Pupilla* often lead to oversplitting (Balashov, Neiber, Bogon, & Hausdorf, 2019; Nekola, Coles, & Horsák, 2015). Old shells (i.e. those whose periostracum was completely eroded) were not considered further because they can lead to distorted estimates of densities when comparing sites with different soil chemistry, strongly influencing their decay rate (Cernohorsky, Horsák, & Cameron, 2010). All uncertain identifications were verified by an expert. Snails from the experimental module (n = 44) were sampled in 2015, while those from the observational module (n = 39) were sampled in 2019. Regional Red-List status in Switzerland according to Rüetschi, Stucki, Müller, Vicentini, & Claude, 2012. LC stands for Least Concern, NT for Near Threatened, VU for Vulnerable, EN for Endangered. No Critically Endangered (CR) or Data Deficient (DD) species were found. Soil moisture preferences were established from the species affinity for each moisture category, extracted from Falkner, Obrdlík, Castella, & Speight (2001).

| Species | Moisture preference | Red-List status | Abundance | |
| --- | --- | --- | --- | --- |
|  |  |  | Experimental module | Observational module |
| *Aegopinella minor* | Mesophilous | LC | 41 | 2 |
| *Aegopinella pura* | Mesophilous | LC | 0 | 10 |
| *Candidula unifasciata ^1^* | Xerophilous | VU | 5 | 84 |
| *Carychium minimum* | Hygrophilous | LC | 81 | 0 |
| *Carychium tridentatum* | Mesophilous | LC | 33 | 1 |
| *Cecilioides acicula ^1,2^* | Mesophilous | LC | 92 | 10 |
| *Cepaea hortensis* | Mesophilous | LC | 0 | 2 |
| *Cepaea nemoralis cf* | Mesophilous | LC | 0 | 4 |
| Species | Moisture preference | Red-List status | Abundance | |
|  |  |  | Experimental module | Observational module |
| *Cochlicopa lubrica ^1^* | Hygrophilous | LC | 55 | 108 |
| *Cochlicopa lubricella ^1,2^* | Mesophilous | LC | 364 | 56 |
| *Columella columella* | Mesophilous | LC | 0 | 1 |
| *Discus rotundatus* | Mesophilous | LC | 1 | 1 |
| *Ena montana* | Mesophilous | LC | 2 | 0 |
| *Euconulus fulvus* | Mesophilous | LC | 21 | 0 |
| *Euomphalia strigella* | Mesophilous | NT | 13 | 0 |
| *Fruticicola fruticum* | Mesophilous | LC | 3 | 0 |
| *Helicella itala* | Xerophilous | NT | 2 | 0 |
| *Jaminia quadridens* | Xerophilous | VU | 0 | 4 |
| *Merdigera obscura* | Mesophilous | LC | 1 | 0 |
| *Nesovitrea hammonis ^2^* | Mesophilous | LC | 58 | 0 |
| *Nesovitrea petronella* | Hygrophilous | LC | 0 | 36 |
| *Oxyloma elegans* | Hygrophilous | NT | 2 | 0 |
| *Platyla polita* | Mesophilous | LC | 16 | 1 |
| *Punctum pygmaeum ^1,2^* | Mesophilous | LC | 335 | 4 |
| *Pupilla muscorum ^1,2^* | Xerophilous | LC | 228 | 623 |
| *Succinella oblonga ^2^* | Mesophilous | LC | 74 | 19 |
| *Trochulus* sp. *^1,2^* | Mesophilous | LC | 245 | 105 |
| *Truncatellina cylindrica ^1,2^* | Xerophilous | LC | 439 | 147 |
| *Vallonia costata ^1,2^* | Xerophilous | LC | 722 | 1490 |
| *Vallonia excentrica ^1,2^* | Xerophilous | LC | 293 | 842 |
| *Vallonia pulchella ^1,2^* | Hygrophilous | LC | 105 | 242 |
| *Vertigo angustior* | Mesophilous | EN | 6 | 1 |
| *Vertigo antivertigo* | Hygrophilous | VU | 0 | 4 |
| *Vertigo pygmaea ^1,2^* | Mesophilous | LC | 297 | 111 |
| *Vitrea contracta* | Mesophilous | LC | 16 | 0 |
| *Vitrina pellucida ^2^* | Mesophilous | LC | 19 | 0 |
| *Xerolenta obvia ^1^* | Xerophilous | NT | 73 | 51 |
| *Zebrina detrita* | Xerophilous | VU | 2 | 1 |
|  |  |  |  |  |
| Not identified |  |  | 62 | 67 |
|  |  |  |  |  |
| *Cochlicopa* sp. |  |  | 26 | 76 |
| *Vallonia* sp. |  |  | 294 | 836 |
| *Vertigo* sp. |  |  | 0 | 18 |

^1^ Species included in the community analysis used to investigate the long-term effects of grassland management intensification (both modules).

^2^ Species included in the community analysis used to investigate the mid-term effects of grassland management intensification (experimental module).

**References**

Balashov, I., Neiber, M. T., Bogon, K., & Hausdorf, B. (2019). On the identity of *Pupilla bigranata* populations from Germany and Ukraine (Gastropoda: Pupillidae). *Archiv Für Molluskenkunde*, *148*, 1–7.

Cernohorsky, N. H., Horsák, M., & Cameron, R. A. D. (2010). Land snail species richness and abundance at small scales: The effects of distinguishing between live individuals and empty shells. *Journal of Conchology*, *40*(2), 233–241.

Falkner, G., Obrdlík, P., Castella, E., & Speight, M. C. D. (2001). *Shelled Gastropoda of Western Europe*. München: Friedrich-Held-Gesellschaft.

Hausser, J. (2005). *Fauna Helvetica: Mollusca Identification*. Neuchâtel: Centre suisse de cartografie de faune (CSCF/SZKF) & Schweizerisches Entomologische Gesellschaft (SEG/SES).

Horsáková, V., Nekola, J. C., & Horsák, M. (2020). Integrative taxonomic consideration of the Holarctic *Euconulus fulvus* group of land snails (Gastropoda, Stylommatophora). *Systematics and Biodiversity*, *18*(2), 142–160. https://doi.org/10.1080/14772000.2020.1725172

Nekola, J. C., Coles, B. F., & Horsák, M. (2015). Species assignment in *Pupilla* (Gastropoda: Pulmonata: Pupillidae): integration of DNA-sequence data and conchology. *Journal of Molluscan Studies*, *81*, 196–216. https://doi.org/10.1093/mollus/eyu083

Rüetschi, J., Stucki, P., Müller, P., Vicentini, H., & Claude, F. (2012). *Liste rouge Mollusques (gastéropodes et bivalves). Espèces menacées en Suisse, état 2010*. *L’environnement pratique, n^o^ 1216*. Office fédéral de l’environnement, Bern, & Centre suisse de cartographie de la faune, Neuchâtel.
