# Supplementary material for: Mid- and long-term responses of land snail communities to the intensification of mountain hay meadows management
Source: BMC Ecol Evol. 2022 Feb 15;22:19. doi: 10.1186/s12862-022-01972-4 (PMC8845342; doi:10.1186/s12862-022-01972-4)
Supplement: Supplementary file 4 — Additional file 4: Appendix S4. Results of the response of snail communities to long-term management intensification (both modules). [file 12862_2022_1972_MOESM4_ESM.docx]

**Mid- and long-term responses of land snail communities to the intensification of mountain hay meadows management**

Gerard Martínez-De León^a, *^, Lauriane Dani^a^, Aline Hayoz-Andrey^a^, Ségolène Humann-Guilleminot^a^, Raphaël Arlettaz^a^ and Jean-Yves Humbert^a^

^a^ Division of Conservation Biology, Institute of Ecology and Evolution, University of Bern, Baltzerstrasse 6, 3012 Bern, Switzerland

*Corresponding author

Email: [gerard.martinezdeleon@iee.unibe.ch](mailto:gerard.martinezdeleon@iee.unibe.ch); [martinezdeleongerard@gmail.com](mailto:martinezdeleongerard@gmail.com)

**Appendix S4 – Results of the response of snail communities to long-term management intensification (both modules)**

| Table S4.1 | Results of the GLMM on overall snail density |
| --- | --- |
| Table S4.2 | Results of the GLMM on overall snail species richness |
| Table S4.3 | Results of the LMM on evenness (Pielou’s index) |
| Table S4.4 | Results of the GLMM on the number of red-listed species |
| Table S4.5 | Results of the GLMM on snail density for each of the moisture preference groups (xerophilous, mesophilous, hygrophilous) |
| Table S4.6 | Results of the GLMM on snail species richness for each of the moisture preference groups (xerophilous, mesophilous, hygrophilous) |
| Table S4.7 | Results of the LMM on the CWM of moisture preferences |
|  |  |
|  |  |
|  |  |
|  |  |
|  |  |

This appendix provides the outputs of the generalised linear mixed-effects models (GLMM), linear mixed-effects models (LMM) and multivariate generalised linear models performed to analyse the response of snail communities to long-term grassland management intensification. Data from the extensive and mid-term intensified managements came from the experimental module, while data from the long-term intensified management are from the observational module. The response variables investigated were density and species richness (overall, and by moisture preference groups), evenness (Pielou’s index), number of red-listed species, community weighted mean (CWM) of moisture preferences and community composition. For the latter, the results on the overall community and the univariate responses of each species are presented. In all the analyses, a subset of samples with soil pH > 6 was considered in order to control the limitation effect of pH on snail communities (see Table S1.1 and S1.3 for a description of soil pH in each meadow). In the GLMM and LMM, post hoc test for multiple comparison analysis were performed using the function *relevel* to set other treatments as intercept. The figures showing relevant results are displayed as well.

Table of content

| Table S4.8 | Results of the multivariate generalised linear model (overall community composition) |
| --- | --- |
| Table S4.9 | Univariate test statistics from the multivariate generalised linear model (species abundances) |
| Fig. S4.1 | Evenness (Pielou’s index) in response to meadow management intensification |
| Fig. S4.2 | CWM of moisture preferences in response to meadow management intensification |
| Fig. S4.3 | Species abundances in response to meadow management intensification |

**Table S4.1.** Output of the GLMM with Poisson distribution used to investigate the effect of long-term management intensification on overall snail density. An observation-level random factor was added to the models to account for overdispersion. Study site was also set as a random factor. Estimates, standard errors (SE) and p-values (*P*) are provided**.**

|  | Snail density (log-scale) | | |
| --- | --- | --- | --- |
| Meadow management | Estimate | SE | *P* |
| Intercept (Extensive) | 3.628 | 0.464 | **< 0.001** |
| Mid-term intensified vs Extensive | 0.913 | 0.401 | **0.023** |
| Long-term intensified vs Extensive | 0.138 | 0.492 | 0.779 |
|  |  |  |  |
| Intercept (Mid-term intensified) | 4.541 | 0.451 | **< 0.001** |
| Long-term intensified vs Mid-term intensified | -0.775 | 0.487 | 0.112 |
|  |  |  |  |
| *Random effects* |  |  |  |
| Observation-level | 0.707 |  |  |
| Site | 1.724 |  |  |

**Table S4.2.** Output of the GLMM with Poisson distribution used to investigate the effect of long-term management intensification on overall snail species richness. Study site was set as a random factor. Estimates, standard errors (SE) and p-values (*P*) are provided**.**

|  | Snail species richness (log-scale) | | |
| --- | --- | --- | --- |
| Meadow management | Estimate | SE | *P* |
| Intercept (Extensive) | 2.096 | 0.162 | **< 0.001** |
| Mid-term intensified vs Extensive | 0.017 | 0.151 | 0.911 |
| Long-term intensified vs Extensive | -0.417 | 0.174 | **0.016** |
|  |  |  |  |
| Intercept (Mid-term intensified) | 2.113 | 0.157 | **< 0.001** |
| Long-term intensified vs Mid-term intensified | -0.434 | 0.171 | **0.011** |
|  |  |  |  |
| *Random effects* |  |  |  |
| Site | 0.174 |  |  |

**Table S4.3.** Output of the LMM used to investigate the effect of long-term management intensification on evenness (Pielou’s index). Study site was set as a random factor. Estimates, standard errors (SE) and p-values (*P*) are provided**.**

|  | Evenness (Pielou’s index) | | |
| --- | --- | --- | --- |
| Meadow management | Estimate | SE | *P* |
| Intercept (Extensive) | 0.881 | 0.032 | **< 0.001** |
| Mid-term intensified vs Extensive | -0.064 | 0.042 | 0.143 |
| Long-term intensified vs Extensive | -0.129 | 0.038 | **0.002** |
|  |  |  |  |
| Intercept (Mid-term intensified) | 0.817 | 0.030 | **< 0.001** |
| Long-term intensified vs Mid-term intensified | -0.065 | 0.037 | 0.088 |
|  |  |  |  |
| *Random effects* |  |  |  |
| Site | 0.001 |  |  |
| Residual | 0.008 |  |  |

**Table S4.4.** Output of the GLMM with Poisson distribution used to investigate the effect of long-term management intensification on the number of red-listed species. Study site was set as a random factor. Estimates, standard errors (SE) and p-values (*P*) are provided**.**

|  | Number of red-listed species (log-scale) | | |
| --- | --- | --- | --- |
| Meadow management | Estimate | SE | *P* |
| Intercept (Extensive) | -1.865 | 0.927 | **0.044** |
| Mid-term intensified vs Extensive | -1.176 | 1.159 | 0.310 |
| Long-term intensified vs Extensive | 0.324 | 0.770 | 0.674 |
|  |  |  |  |
| Intercept (Mid-term intensified) | -3.040 | 1.222 | **0.013** |
| Long-term intensified vs Mid-term intensified | 1.500 | 1.122 | 0.181 |
|  |  |  |  |
| *Random effects* |  |  |  |
| Site | 1.487 |  |  |

**Table S4.5.** Output of the GLMM with Poisson distribution used to investigate the effect of long-term management intensification on snail density for each of the moisture preference groups: xerophilous, mesophilous and hygrophilous. An observation-level random factor was added to the models to account for overdispersion. Study site was also set as a random factor. Estimates, standard errors (SE) and p-values (*P*) are provided**.**

|  | Meadow management | Estimate | SE | *P* |
| --- | --- | --- | --- | --- |
| Density of xerophilous  (log-scale) | Intercept (Extensive) | 2.668 | 0.477 | **< 0.001** |
|  | Mid-term intensified vs Extensive | 0.951 | 0.434 | **0.028** |
|  | Long-term intensified vs Extensive | 0.821 | 0.516 | 0.112 |
|  |  | 2.668 | 0.477 |  |
|  | Intercept (Mid-term intensified) | 3.619 | 0.460 | **< 0.001** |
|  | Long-term intensified vs Mid-term intensified | -0.130 | 0.506 | 0.797 |
|  |  |  |  |  |
|  | *Random effects* |  |  |  |
|  | Observation-level | 0.711 |  |  |
|  | Site | 1.618 |  |  |
| Density of mesophilous  (log-scale) | Intercept (Extensive) | 2.740 | 0.477 | **< 0.001** |
|  | Mid-term intensified vs Extensive | 0.879 | 0.535 | 0.101 |
|  | Long-term intensified vs Extensive | -1.487 | 0.594 | **0.012** |
|  |  |  |  |  |
|  | Intercept (Mid-term intensified) | 3.618 | 0.452 | **< 0.001** |
|  | Long-term intensified vs Mid-term intensified | -2.366 | 0.583 | **< 0.001** |
|  |  |  |  |  |
|  | *Random effects* |  |  |  |
|  | Observation-level | 1.256 |  |  |
|  | Site | 0.811 |  |  |
| Density of hygrophilous  (log-scale) | Intercept (Extensive) | -0.456 | 0.946 | 0.629 |
|  | Mid-term intensified vs Extensive | 0.903 | 0.947 | 0.340 |
|  | Long-term intensified vs Extensive | 0.740 | 1.045 | 0.479 |
|  |  |  |  |  |
|  | Intercept (Mid-term intensified) | 0.446 | 0.870 | 0.608 |
|  | Long-term intensified vs Mid-term intensified | -0.162 | 1.032 | 0.875 |
|  |  |  |  |  |
|  | *Random effects* |  |  |  |
|  | Observation-level | 2.538 |  |  |
|  | Site | 3.435 |  |  |

**Table S4.6.** Output of the GLMM with Poisson distribution used investigate the effect of long-term management intensification on snail species richness for each of the moisture preference groups: xerophilous, mesophilous and hygrophilous. Study site was set as a random factor. Estimates, standard errors (SE) and p-values (*P*) are provided**.**

|  | Meadow management | Estimate | SE | *P* |
| --- | --- | --- | --- | --- |
| Richness of xerophilous  (log-scale) | Intercept (Extensive) | 1.144 | 0.208 | **< 0.001** |
|  | Mid-term intensified vs Extensive | 0.083 | 0.247 | 0.737 |
|  | Long-term intensified vs Extensive | 0.057 | 0.241 | 0.812 |
|  |  |  |  |  |
|  | Intercept (Mid-term intensified) | 1.227 | 0.192 | **< 0.001** |
|  | Long-term intensified vs Mid-term intensified | -0.025 | 0.229 | 0.911 |
|  |  |  |  |  |
|  | *Random effects* |  |  |  |
|  | Site | 0.083 |  |  |
| Richness of mesophilous  (log-scale) | Intercept (Extensive) | 1.420 | 0.219 | **< 0.001** |
|  | Mid-term intensified vs Extensive | -0.088 | 0.205 | 0.667 |
|  | Long-term intensified vs Extensive | -0.964 | 0.243 | **0.007** |
|  |  |  |  |  |
|  | Intercept (Mid-term intensified) | 1.332 | 0.216 | **< 0.001** |
|  | Long-term intensified vs Mid-term intensified | -0.876 | 0.244 | **< 0.001** |
|  |  |  |  |  |
|  | *Random effects* |  |  |  |
|  | Site | 0.283 |  |  |
| Richness of hygrophilous  (log-scale) | Intercept (Extensive) | -0.584 | 0.457 | 0.201 |
|  | Mid-term intensified vs Extensive | 0.324 | 0.529 | 0.540 |
|  | Long-term intensified vs Extensive | 0.390 | 0.497 | 0.433 |
|  |  |  |  |  |
|  | Intercept (Mid-term intensified) | -0.260 | 0.385 | 0.500 |
|  | Long-term intensified vs Mid-term intensified | 0.066 | 0.441 | 0.881 |
|  |  |  |  |  |
|  | *Random effects* |  |  |  |
|  | Site | 0.301 |  |  |

**Table S4.7.** Output of the LMM used to investigate the effect of long-term management intensification on the community weighted mean (CWM) of moisture preference. Values range from 1 (preference for dry habitats) to 3 (preference for wet habitats). Study site was set as a random factor. Estimates, standard errors (SE) and p-values (*P*) are provided**.**

|  | CWM of moisture preference | | |
| --- | --- | --- | --- |
| Meadow management | Estimate | SE | *P* |
| Intercept (Extensive) | 1.618 | 0.098 | **< 0.001** |
| Mid-term intensified vs Extensive | -0.018 | 0.101 | 0.857 |
| Long-term intensified vs Extensive | -0.262 | 0.113 | **0.026** |
|  |  |  |  |
| Intercept (Mid-term intensified) | 1.599 | 0.094 | **< 0.001** |
| Long-term intensified vs Mid-term intensified | -0.243 | 0.110 | **0.034** |
|  |  |  |  |
| *Random effects* |  |  |  |
| Site | 0.051 |  |  |
| Residual | 0.047 |  |  |

**Table S4.8.** Output of the multivariate generalised linear model with negative binomial distribution performed to investigate the effect of long-term management intensification on community composition, based on species abundances. Study site was also added as a fixed factor in the model. Species included in the analysis are listed in Table S2.1 and Table S4.9. The function *anova.manyglm* in the package *mvabund* (Wang, Naumann, Eddelbuettel, Wilshire, & Warton, 2020) was used to compute the analysis of deviance table for the model fit. Likelihood-ratio values were summed across all species to get a statistic for the whole community. P-values were calculated using 999 iterations via PIT-trap resampling. Values with *P* < 0.05 are marked in bold.

|  | Deviance | Df of residuals | *P* |
| --- | --- | --- | --- |
| (Intercept) |  | 41 |  |
| Meadow management | 75.8 | 39 | **0.003** |
| Site | 574.9 | 23 | **0.001** |

**Table S4.9**. Univariate test statistics from the multivariate generalised linear models with negative binomial distribution performed to investigate the effect of long-term management intensification on community composition, based on snail species abundances. The function *anova.manyglm* in the package *mvabund* (Wang, Naumann, Eddelbuettel, Wilshire, & Warton, 2020) was used to compute the analysis of deviance table for the model fit. P-values were calculated using 999 iterations via PIT-trap resampling and adjusted for multiple testing. Values with *P* < 0.05 are marked in bold.

| Species |  | Meadow management | Site |
| --- | --- | --- | --- |
| *Candidula unifasciata* | Deviance | 5.127 | 51.852 |
|  | *P* | 0.542 | **0.045** |
| *Cecilioides acicula* | Deviance | 3.443 | 43.330 |
|  | *P* | 0.763 | 0.115 |
| *Cochlicopa lubrica* | Deviance | 1.724 | 25.178 |
|  | *P* | 0.835 | 0.370 |
| *Cochlicopa lubricella* | Deviance | 13.842 | 47.419 |
|  | *P* | **0.029** | **0.071** |
| *Punctum pygmaeum* | Deviance | 14.213 | 39.220 |
|  | *P* | **0.026** | 0.158 |
| *Pupilla muscorum* | Deviance | 11.683 | 48.472 |
|  | *P* | 0.056 | **0.071** |
| *Trochulus* sp. | Deviance | 2.316 | 49.299 |
|  | *P* | 0.835 | **0.071** |
| *Truncatellina cylindrica* | Deviance | 2.123 | 32.710 |
|  | *P* | 0.835 | 0.274 |
| *Vallonia costata* | Deviance | 8.840 | 60.615 |
|  | *P* | 0.164 | **0.008** |
| *Vallonia excentrica* | Deviance | 7.671 | 47.459 |
|  | *P* | 0.210 | **0.071** |
| *Vallonia pulchella* | Deviance | 3.297 | 47.626 |
|  | *P* | 0.763 | **0.071** |
| *Vertigo pygmaea* | Deviance | 1.306 | 40.968 |
|  | *P* | 0.835 | 0.158 |
| *Xerolenta obvia* | Deviance | 0.217 | 40.726 |
|  | *P* | 0.835 | 0.158 |

**
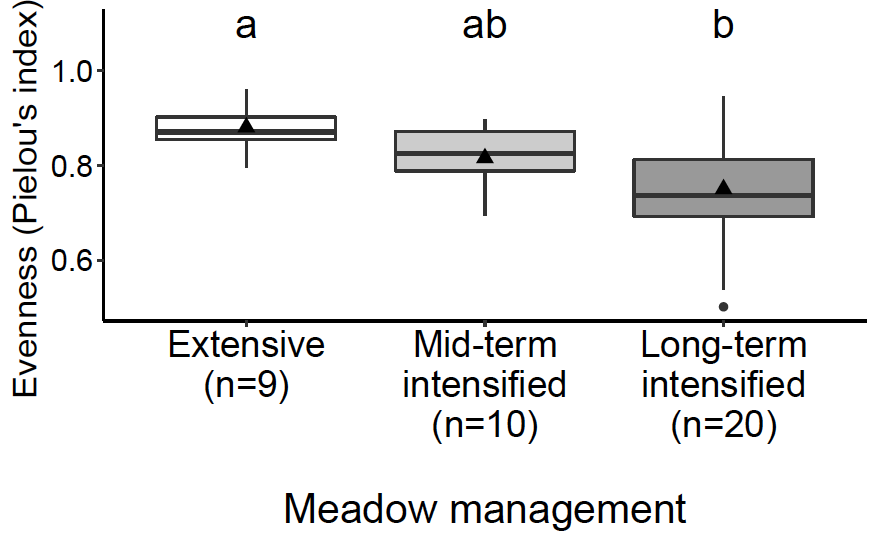
**

**Fig. S4.1.** Effect of meadow management intensification on snail evenness (Pielou’s index). Data for the extensive (no water and fertiliser inputs, i.e. control plots) and mid-term intensive (plots having received high inputs of water and fertiliser during five years) management types stemmed from the experimental module, whereas data from the long-term intensive management (> 20 years) are drawn from the observational module. Bold lines represent box-plot medians, solid triangles means, boxes the first and third quantiles, whiskers the inter-quartile distance multiplied by 1.5, and solid dots the outliers. Different letters indicate significant differences between treatments at *P* < 0.05.


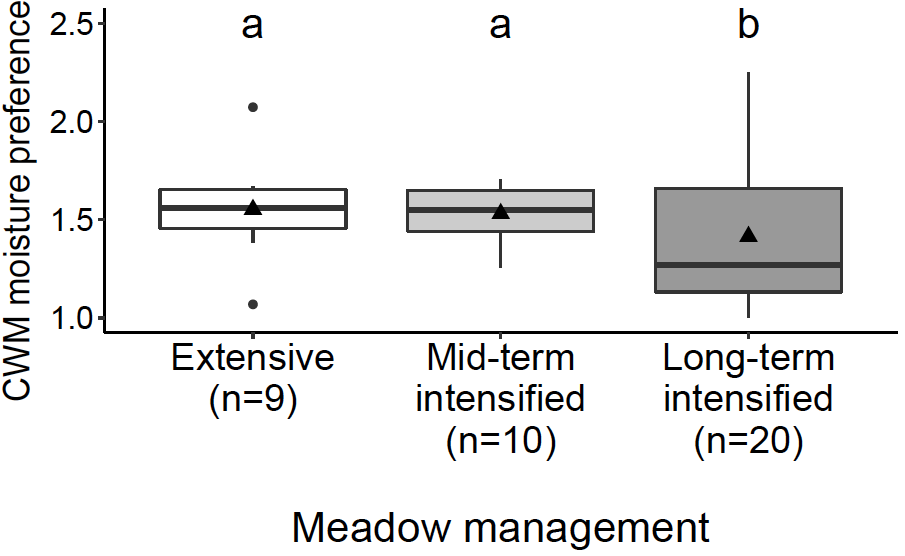


**Fig. S4.2.** Effect of meadow management intensification on the community weighted mean (CWM) of moisture preferences, on a scale ranging from 1 (xerophilous community) to 3 (hygrophilous community). For management descriptions and box-plot features, see legend of Fig. S4.1.


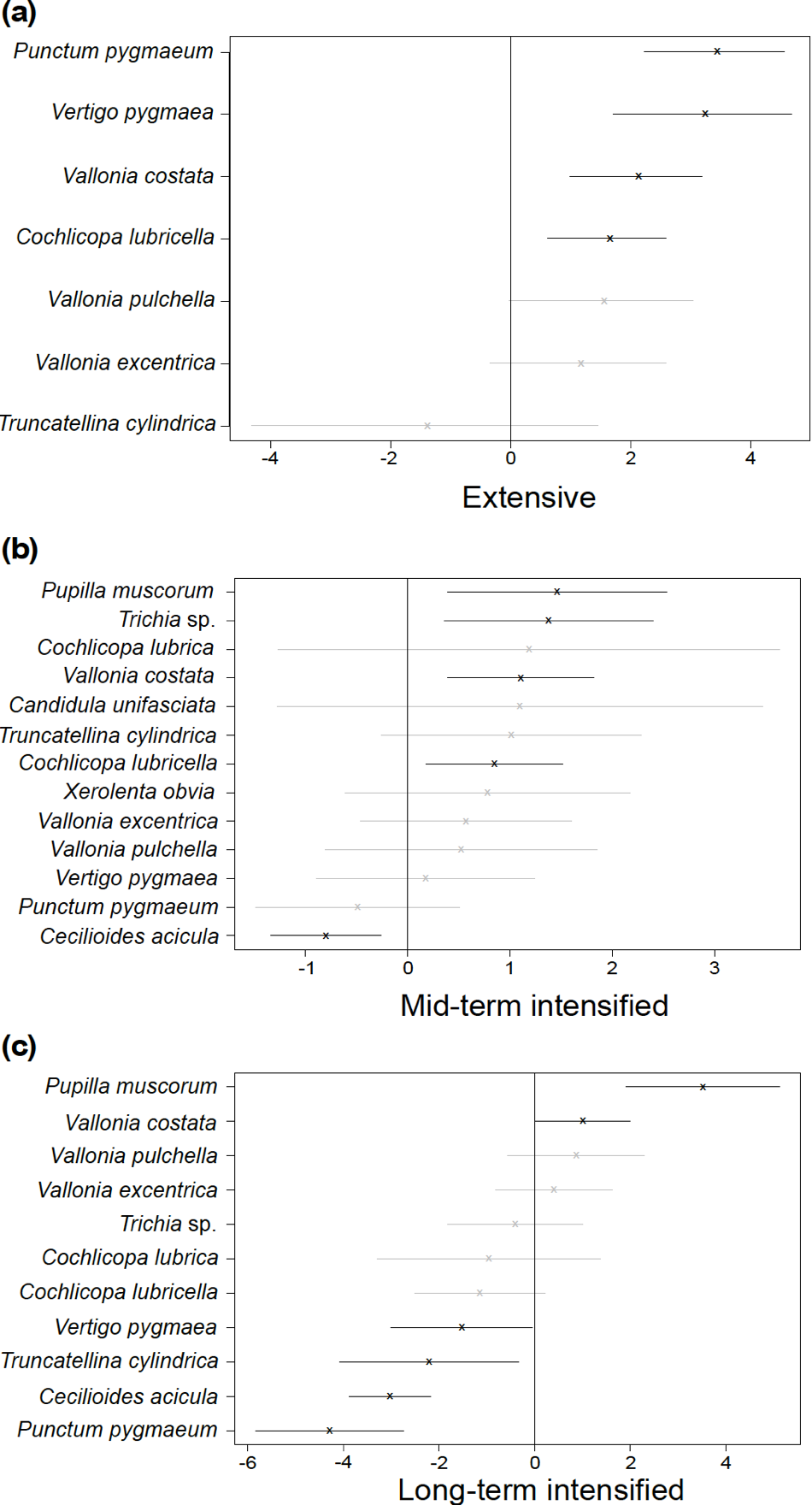


**Fig. S4.3.** Plots of the point estimates for the coefficients of the model-based community analysis: a) extensive meadow management; b) mid-term intensified; c) long-term intensified. Bars show 95% confidence intervals, with those coloured in black indicating intervals not containing zero. Species showing significant responses are shown in Table S4.9 (p-values calculated with permutational methods and corrected for multiple testing). Species with very large confidence intervals are not displayed for visualisation purposes.

**References**

Wang, Y., Naumann, U., Eddelbuettel, D., Wilshire, J., & Warton, D. (2020). mvabund: Statistical Methods for Analysing Multivariate Abundance Data. R package version 4.1.3. Retrieved from https://cran.r-project.org/package=mvabund
