# Supplementary material for: Mid- and long-term responses of land snail communities to the intensification of mountain hay meadows management
Source: BMC Ecol Evol. 2022 Feb 15;22:19. doi: 10.1186/s12862-022-01972-4 (PMC8845342; doi:10.1186/s12862-022-01972-4)
Supplement: Supplementary file 5 — Additional file 5: Appendix S5. Results of the experimental module. [file 12862_2022_1972_MOESM5_ESM.docx]

**Mid- and long-term responses of land snail communities to the intensification of mountain hay meadows management**

Gerard Martínez-De León^a, *^, Lauriane Dani^a^, Aline Hayoz-Andrey^a^, Ségolène Humann-Guilleminot^a^, Raphaël Arlettaz^a^ and Jean-Yves Humbert^a^

^a^ Division of Conservation Biology, Institute of Ecology and Evolution, University of Bern, Baltzerstrasse 6, 3012 Bern, Switzerland

*Corresponding author

Email: [gerard.martinezdeleon@iee.unibe.ch](mailto:gerard.martinezdeleon@iee.unibe.ch); [martinezdeleongerard@gmail.com](mailto:martinezdeleongerard@gmail.com)

**Appendix S5 – Results of the experimental module**

This appendix provides the outputs of the generalised linear mixed-effects models (GLMM), linear mixed-effects models (LMM) and multivariate generalised linear models used to investigate the mid-term effect (after five years) of management intensification (4-levels management intensity gradient consisting of control (no input), low-, medium- and high-input levels) on snail communities and soil pH. The variables used to measure the effect on snail communities were density and species richness (overall, as well as on xerophilous and mesophilous snails), evenness and community composition. For the latter, the results on the overall community and the univariate responses of each species are presented. The figures showing relevant results are displayed as well.

Table of content

| Table S5.1 | Results of the GLMM on overall snail density and snail species richness |
| --- | --- |
| Table S5.2 | Results of the LMM on evenness (Pielou’s index) |
| Table S5.3 | Results of the GLMM on densities of xerophilous and mesophilous snails |
| Table S5.4 | Results of the GLMM on species richness of xerophilous and mesophilous snails |
| Table S5.5 | Results of the LMM on soil pH |
| Table S5.6 | Results of the multivariate generalised linear model (overall community composition) |
| Table S5.7 | Univariate test statistics from the multivariate generalised linear model (species abundances) |
| Fig. S5.1 | Evenness (Pielou’s index) in response to the management intensity gradient |
| Fig. S5.2 | Densities of xerophilous and mesophilous snails in response to the management intensity gradient |
| Fig. S5.3 | Soil pH in response to the management intensity gradient |
| Fig. S5.4 | Species abundances in response to the management intensity gradient |

**Table S5.1**. Output of the GLMM with Poisson distribution to measure the impact of mid-term management intensification on snail density and snail species richness. Estimates, standard errors (SE) and p-values (*P*) are provided. Significant p*-*values are highlighted in bold. “Intercept” represents the intercept of the regression; “Management intensity” represents the slope of the regression. An observation-level random factor was added to the models to account for overdispersion. Study site was also set as a random factor.

|  |  | Estimate | SE | *P* |
| --- | --- | --- | --- | --- |
| Snail density  (log-scale) | Intercept | 3.537 | 0.270 | **< 0.001** |
|  | Management intensity | 0.328 | 0.082 | **< 0.001** |
|  |  |  |  |  |
|  | *Random effects* |  |  |  |
|  | Observation-level | 0.337 |  |  |
|  | Site | 0.538 |  |  |
| Snail species richness  (log-scale) | Intercept | 2.086 | 0.112 | **< 0.001** |
|  | Management intensity | 0.031 | 0.046 | 0.491 |
|  |  |  |  |  |
|  | *Random effects* |  |  |  |
|  | Site | 0.052 |  |  |

**Table S5.2**. Output of the LMM used to measure the impact of mid-term management intensification on evenness (Pielou’s index). Estimates, standard errors (SE) and p-values (*P*) are provided. Significant p*-*values are highlighted in bold. “Intercept” represents the intercept of the regression; “Management intensity” represents the slope of the regression. Study site was set as a random factor.

|  | Evenness | | |
| --- | --- | --- | --- |
|  | Estimate | SE | *P* |
| Intercept | 0.854 | 0.020 | **< 0.001** |
| Management intensity | -0.017 | 0.008 | **0.039** |
|  |  |  |  |
| *Random effects* |  |  |  |
| Site | 0.002 |  |  |
| Residual | 0.004 |  |  |

**Table S5.3.** Outputs of the GLMM with Poisson distribution used to measure the impact of mid-term management intensification on the density of xerophilous and mesophilous snails. Estimates, standard errors (SE) and p-values (*P*) are provided**.** Significant p*-*values are highlighted in bold. “Intercept” represents the intercept of the regression; “Management intensity” represents the slope of the regression. An observation-level random factor was added to the models to account for overdispersion. Study site was also set as a random factor.

|  |  | Estimate | SE | *P* |
| --- | --- | --- | --- | --- |
| Density of xerophilous  (log-scale) | Intercept | 3.218 | 0.203 | **< 0.001** |
|  | Management intensity | 0.250 | 0.074 | **< 0.001** |
|  |  |  |  |  |
|  | *Random effects* |  |  |  |
|  | Observation-level | 0.264 |  |  |
|  | Site | 0.237 |  |  |
| Density of mesophilous  (log-scale) | Intercept | 2.386 | 0.318 | **< 0.001** |
|  | Management intensity | 0.401 | 0.100 | **< 0.001** |
|  |  |  |  |  |
|  | *Random effects* |  |  |  |
|  | Observation-level | 0.463 |  |  |
|  | Site | 0.703 |  |  |

**Table S5.4.** Outputs of the GLMM with Poisson distribution used to measure the impact of mid-term management intensification on the richness of xerophilous and mesophilous snail species. Estimates, standard errors (SE) and p-values (*P*) are provided. Significant p*-*values are highlighted in bold. “Intercept” represents the intercept of the regression; “Management intensity” represents the slope of the regression Estimates, standard errors (SE) and p-values (*P*) are provided**.** Study site was set as a random factor.

|  |  | Estimate | SE | *P* |
| --- | --- | --- | --- | --- |
| Richness of xerophilous  (log-scale) | Intercept | 1.464 | 0.127 | **< 0.001** |
|  | Management intensity | 0.028 | 0.063 | 0.658 |
|  |  |  |  |  |
|  | *Random effects* |  |  |  |
|  | Site | 0.018 |  |  |
| Richness of mesophilous  (log-scale) | Intercept | 1.407 | 0.169 | **< 0.001** |
|  | Management intensity | 0.010 | 0.063 | 0.873 |
|  |  |  |  |  |
|  | *Random effects* |  |  |  |
|  | Site | 0.069 |  |  |

Table S5.5. Output of the LMM used to assess the relationship between mid-term management intensification and soil pH. Estimates, standard errors (SE) and p-values (*P*) are provided. Significant p*-*values are highlighted in bold. “Intercept” represents the intercept of the regression; “Management intensity” represents the slope of the regression. Study site was set as a random factor.

|  | pH | | |
| --- | --- | --- | --- |
|  | Estimate | SE | *P* |
| Intercept | 6.673 | 0.193 | **< 0.001** |
| Management intensity | 0.109 | 0.042 | **0.014** |
|  |  |  |  |
| *Random effects* |  |  |  |
| Site | 0.341 |  |  |
| Residual | 0.096 |  |  |

**Table S5.6.** Output of the multivariate generalised linear model with negative binomial distribution performed to investigate the effect of mid-term management intensification (continuous variable) on community composition, based on species abundances. Study site was also added as a fixed factor in the model. Species included in the analysis are listed in Table S3.1 and Table S5.7. The function *anova.manyglm* in the package *mvabund* (Wang, Naumann, Eddelbuettel, Wilshire, & Warton, 2020) was used to compute the analysis of deviance table for the model fit. Likelihood-Ratio values were summed across all species to get a statistic for the whole community. P-values were calculated using 999 iterations via PIT-trap resampling. Values with *P* < 0.05 are marked in bold.

|  | Deviance | Df of residuals | *P* |
| --- | --- | --- | --- |
| (Intercept) |  | 43 |  |
| Management intensity | 38.7 | 42 | **0.026** |
| Site | 606.6 | 32 | **< 0.001** |

**Table S5.7**. Univariate test statistics from the multivariate generalised linear model with negative binomial distribution performed to investigate the effect of mid-term management intensification (continuous variable) on community composition, based on species abundances. The function *anova.manyglm* in the package *mvabund* (Wang, Naumann, Eddelbuettel, Wilshire, & Warton, 2020) was used to compute the analysis of deviance table for the model fit. P-values were calculated using 999 iterations via PIT-trap resampling and adjusted for multiple testing. Values with *P* < 0.05 are marked in bold.

| Species |  | Management | Site |
| --- | --- | --- | --- |
| *Cecillioides acicula* | Deviance | 0.465 | 58.747 |
|  | *P* | 0.915 | **0.001** |
| *Cochlicopa lubricella* | Deviance | 7.801 | 27.153 |
|  | *P* | 0.100 | 0.070 |
| *Nesovitrea hammonis* | Deviance | 1.903 | 25.895 |
|  | *P* | 0.702 | 0.070 |
| *Punctum pygmaeum* | Deviance | 0.336 | 58.982 |
|  | *P* | 0.918 | **0.001** |
| *Pupilla muscorum* | Deviance | 4.536 | 31.905 |
|  | *P* | 0.308 | **0.027** |
| *Succinella oblonga* | Deviance | 0.855 | 21.361 |
|  | *P* | 0.848 | 0.199 |
| *Trochulus* sp*.* | Deviance | 3.545 | 58.460 |
|  | *P* | 0.458 | **0.001** |
| *Truncatellina cylindrica* | Deviance | 3.052 | 38.058 |
|  | *P* | 0.477 | **0.006** |
| *Vallonia costata* | Deviance | 9.556 | 45.358 |
|  | *P* | 0.058 | **0.002** |
| *Vallonia excentrica* | Deviance | 1.997 | 55.622 |
|  | *P* | 0.702 | **0.001** |
| *Vallonia pulchella* | Deviance | 0.962 | 45.725 |
|  | *P* | 0.846 | **0.002** |
| *Vertigo pygmaea* | Deviance | 3.552 | 61.756 |
|  | *P* | 0.458 | **0.001** |
| *Vitrina pellucida* | Deviance | 0.050 | 21.700 |
|  | *P* | 0.968 | 0.199 |


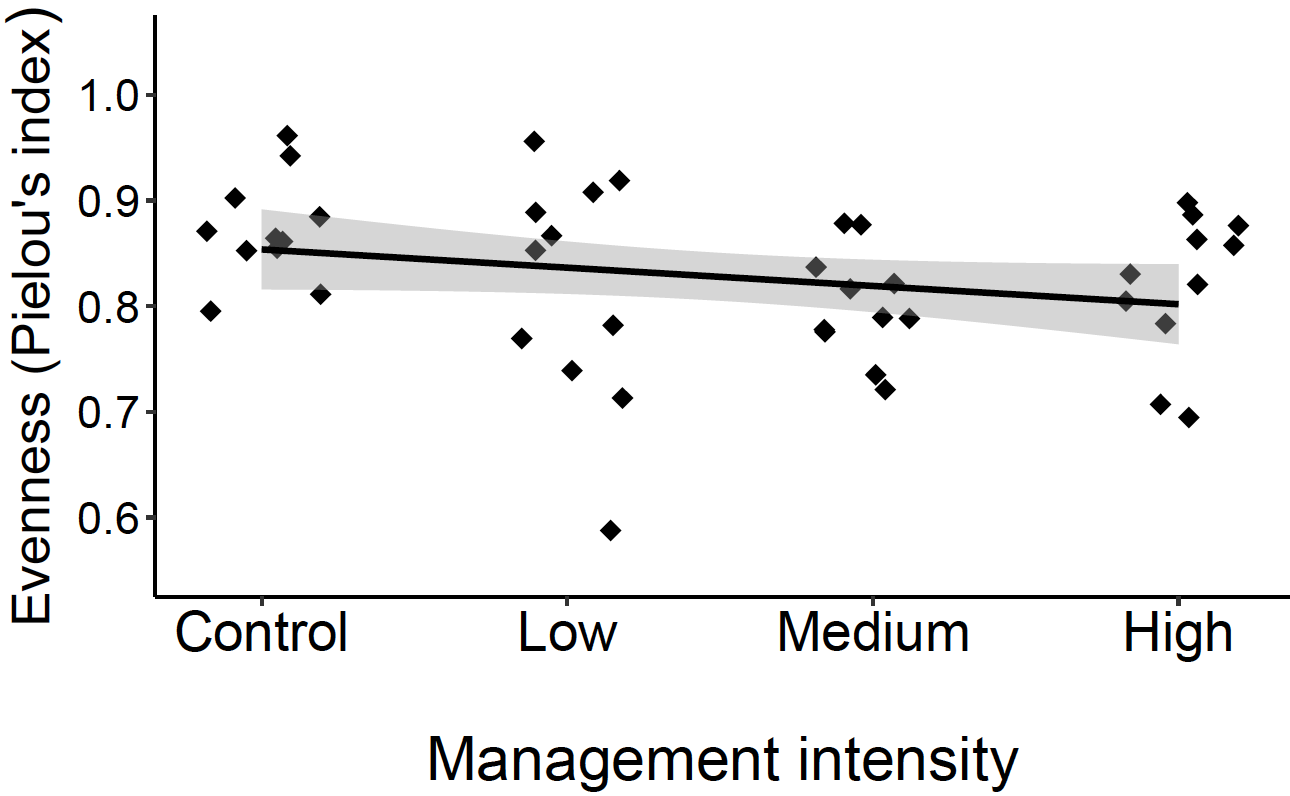


**Fig. S5.1.** Evenness (Pielou’s index) in response to mid-term management intensification, represented by a 4-level intensity gradient consisting of control (no input), low-, medium- and high-input levels. The black line represents the fitted model, along with a 95% confidence band in grey. For further details of the model outputs, see Table S5.2.


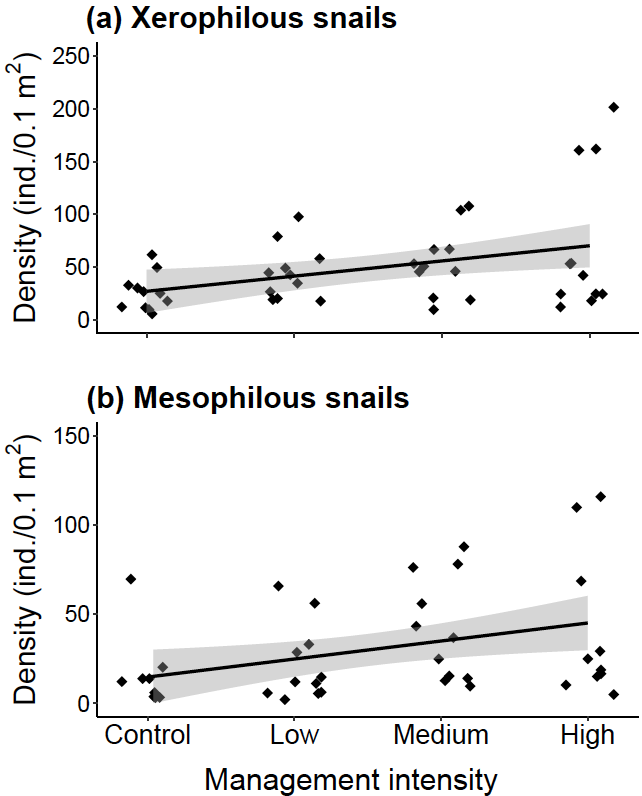


**Fig. S5.2.** Density of snails in response to mid-term management intensification, classified according to their moisture preferences: (a) xerophilous and (b) mesophilous snails. The 4-level management intensity gradient consisted of control (no input), low-, medium- and high-input levels. The black line represents the fitted model, along with a 95% confidence band in grey. For further details of the model outputs, see Table S5.3.


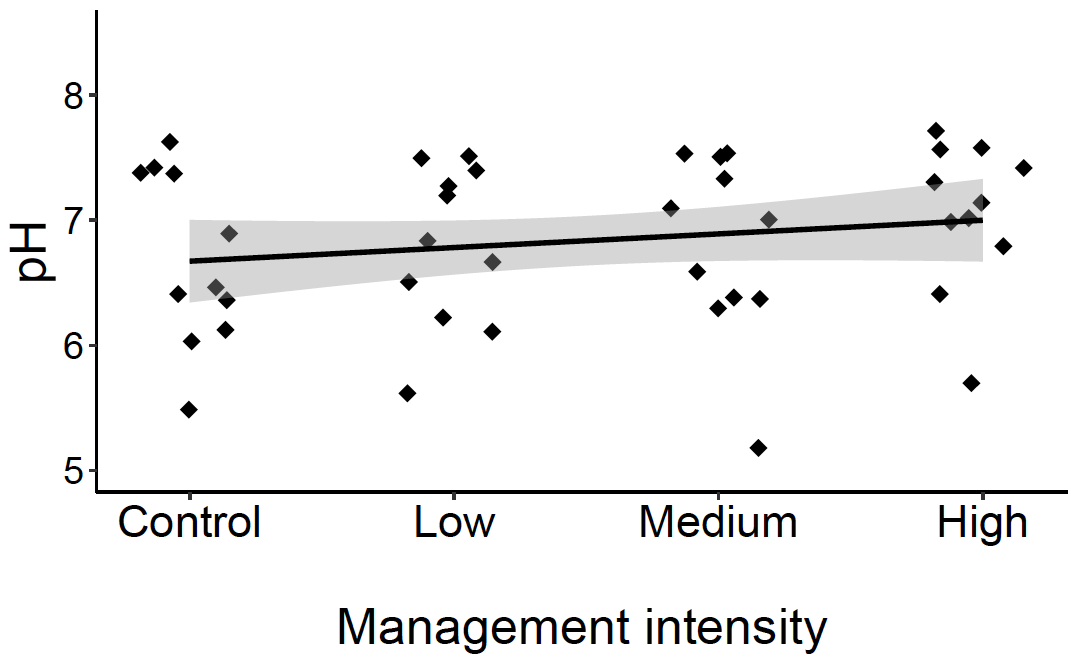


**Fig. S5.3.** Soil pH in response to response to mid-term management intensification, represented by a 4-level intensity gradient consisting of control (no input), low-, medium- and high-input levels. The black line represents the fitted model, along with a 95% confidence band in grey. For further details of the model outputs, see Table S5.5.

**
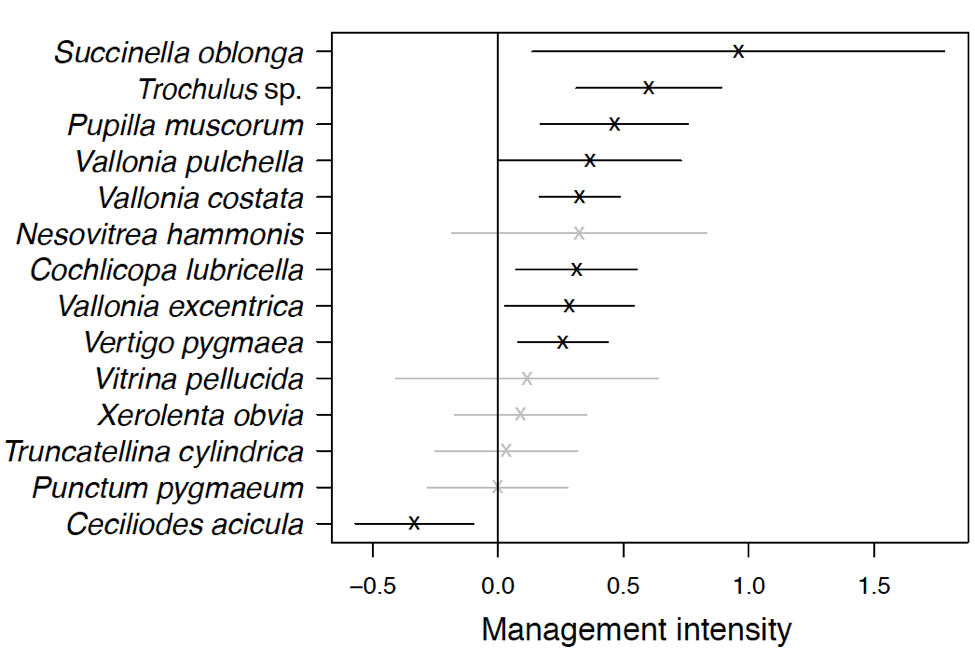
**

**Fig. S5.4.** Plots of the point estimates of the model-based community analysis, representing the response of species abundances to mid-term management intensification. The bars show 95% confidence intervals, with those coloured in black indicating intervals not containing zero. Species showing significant responses are shown in Table S5.7 (p-values calculated with permutational methods and corrected for multiple testing).
