# Supplementary material for: Mid- and long-term responses of land snail communities to the intensification of mountain hay meadows management
Source: BMC Ecol Evol. 2022 Feb 15;22:19. doi: 10.1186/s12862-022-01972-4 (PMC8845342; doi:10.1186/s12862-022-01972-4)
Supplement: Supplementary file 6 — Additional file 6: Appendix S6. Results of the observational module. [file 12862_2022_1972_MOESM6_ESM.docx]

**Mid- and long-term responses of land snail communities to the intensification of mountain hay meadows management**

Gerard Martínez-De León^a, *^, Lauriane Dani^a^, Aline Hayoz-Andrey^a^, Ségolène Humann-Guilleminot^a^, Raphaël Arlettaz^a^ and Jean-Yves Humbert^a^

^a^ Division of Conservation Biology, Institute of Ecology and Evolution, University of Bern, Baltzerstrasse 6, 3012 Bern, Switzerland

*Corresponding author

Email: [gerard.martinezdeleon@iee.unibe.ch](mailto:gerard.martinezdeleon@iee.unibe.ch); [martinezdeleongerard@gmail.com](mailto:martinezdeleongerard@gmail.com)

**Appendix S6 – Results of the observational module**

The key variables shaping snail communities in the meadows of the observational module (long-term effects of intensive management) were identified using a model selection approach. This appendix provides the output of the best set of generalised linear mixed-effects models (GLMM) that were incorporated for model selection and averaging. Model selection could not be performed with the number of red-listed species as a response variable given that their scarce occurrence in the study sites impeded doing so. Instead, the variables having significant effects (*P* < 0.05) in univariate generalised linear mixed models are presented.

Table of content

| Table S6.1 | Output of the GLMM (best set of models with ∆ AICc < 6) performed to analyse the effect of explanatory variables on snail density and species richness |
| --- | --- |
| Table S6.2 | Output of the full model averaging performed on the best set of models (∆ AICc < 6) analysing the effect of explanatory variables on snail density |
| Table S6.3 | Output of the full model averaging performed on the best set of models (∆ AICc < 6) analysing the effect of explanatory variables on snail species richness |
| Table S6.4 | Output of the univariate GLMM analysing the effect of explanatory variables on the number of red-listed species |

**Table S6.1**. GLMM outputs performed to analyse the effect of explanatory variables on snail density and snail species richness. Models were run with Poisson error distribution. The table shows the best set of models (∆ AICc < 6) retained for model averaging. Explanatory variables were first pre-selected from the whole set of variables with univariate GLMMs (see Table S2.1). Those with significant effects (*P* < 0.05) were used for model selection. Interactions with soil pH and any of the other pre-selected explanatory variables were tested and incorporated in the analysis, providing statistical significance. Likewise, polynomial relationships were only considered in case they had significant effects.

| Rank | Model | Df | logLik | AICc | ∆ AICc | Model weight |
| --- | --- | --- | --- | --- | --- | --- |
|  | **Snail density** (with observation-level random effect) | | | | | |
| 1 | bare ground + pH: plant diversity + poly(elevation,2) | 9 | -155.75 | 335.70 | 0.00 | 0.39 |
| 2 | pH : plant diversity + poly(elevation,2) | 8 | -157.81 | 336.40 | 0.72 | 0.27 |
| 3 | bare ground + pH + plant diversity + poly(elevation,2) | 8 | -157.83 | 336.50 | 0.76 | 0.26 |
| 4 | pH + plant diversity + poly(elevation,2) | 7 | -160.59 | 338.80 | 3.10 | 0.08 |
|  | **Snail species richness** |  |  |  |  |  |
| 1 | bare ground + pH + plant diversity + poly(elevation,2) | 7 | -68.18 | 154.00 | 0.00 | 0.64 |
| 2 | pH + plant diversity + poly(elevation,2) | 6 | -70.87 | 156.40 | 2.38 | 0.19 |
| 3 | bare ground + pH + poly(elevation,2) | 6 | -71.14 | 156.90 | 2.93 | 0.15 |

**Table S6.2**. Output of the full model averaging performed on the best set of models (∆ AICc < 6) analysing the effect of explanatory variables on snail density (see Table S6.1). Statistically significant variables (*P* < 0.05) are marked in bold. Relative importance (Rel. importance) was calculated by summing up all Akaike weights of the models in the best set where the predictor variable occurs. Variables were standardised (mean = 0, SD = 1).

| Fixed effects | Estimate | 95% CI | *P* | Rel. importance |
| --- | --- | --- | --- | --- |
|  |  |  |  |  |
| (**Intercept**) | 2.647 | (2.109, 3.185) | **< 0.001** |  |
| Bare ground | 0.194 | (-0.163, 0.551) | 0.287 | 0.65 |
| **pH** | 1.454 | (0.985, 1.923) | **< 0.001** | 1.00 |
| **Plant diversity** | 0.581 | (0.253, 0.910) | **< 0.001** | 1.00 |
| **Elevation** | -9.306 | (-14.213, -4.400) | **< 0.001** | 1.00 |
| **Elevation^2^** | -5.494 | (-10.338, -0.649) | **0.026** | 1.00 |
| Plant diversity : pH | 0.368 | (-0.297, 1.032) | 0.279 | 0.65 |

**Table S6.3**. Output of the full model averaging performed on the best set of models (∆ AICc < 6) analysing the effect of explanatory variables on snail species richness (see Table S6.1). Statistically significant variables (*P* < 0.05) are marked in bold. Relative importance (Rel. importance) was calculated by summing up all Akaike weights of the models in the best set where the predictor variable occurs. Variables were standardised (mean = 0, SD = 1).

| Fixed effects | Estimate | 95% CI | *P* | Rel. importance |
| --- | --- | --- | --- | --- |
|  |  |  |  |  |
| (**Intercept**) | 0.957 | (0.655, 1.260) | **< 0.001** |  |
| Bare ground | 0.186 | (-0.067, 0.440) | 0.150 | 0.85 |
| **pH** | 0.713 | (0.463, 0.964) | **< 0.001** | 1.00 |
| Plant diversity | 0.198 | (-0.052, 0.448) | 0.121 | 0.85 |
| **Elevation** | -4.735 | (-7.392, -2.078) | **< 0.001** | 1.00 |
| **Elevation^2^** | -3.617 | (-6.001, -1.233) | **0.003** | 1.00 |

**Table S6.4**. Output of the univariate GLMM analysing the effect of explanatory variables on the number of red-listed species. Variables are ranked according to their absolute estimates (log scale). Statistically significant variables (*P* < 0.05) are marked in bold.

| Fixed effects | Estimate | SE | *P* |
| --- | --- | --- | --- |
| **pH** | 3.472 | 1.549 | **0.025** |
| **Bare ground** | 0.270 | 0.117 | **0.021** |
